# Autoantibody signatures defined by serological proteome analysis in sera from patients with cholangiocarcinoma

**DOI:** 10.1186/s12967-015-0751-2

**Published:** 2016-01-16

**Authors:** Mohammad Zahid Mustafa, Viet Hung Nguyen, François Le Naour, Eleonora De Martin, Elvire Beleoken, Catherine Guettier, Catherine Johanet, Didier Samuel, Jean-Charles Duclos-Vallee, Eric Ballot

**Affiliations:** Inserm, Unité 1193, Université Paris-Saclay, 94800 Villejuif, France; Univ Paris-Sud, UMR-S 1193, Université Paris-Saclay, 94800 Villejuif, France; CASVAB, University of Balochistan, Quetta, Pakistan; Département d’Immunologie Biologique, Unité d’autoimmunité, AP-HP Hôpital Saint Antoine, 75012 Paris, France; Centre Hépato-Biliaire, AP-HP Hôpital Paul-Brousse, 94800 Villejuif, France; DHU Hepatinov, 94800 Villejuif, France; UFR 967 Faculté de Médecine, Université Pierre et Marie Curie, 75006 Paris, France; Laboratoire Anatomie Pathologique, AP-HP Hôpital Bicêtre, 94270 Le Kremlin-Bicêtre, France

**Keywords:** Autoantigens, Autoantibodies, Cholangiocarcinoma, Mass spectrometry, Proteomics

## Abstract

**Background:**

The challenging diagnosis and poor prognosis of cholangiocarcinoma require the determination of biomarkers. Autoantibodies could be used in the clinic as diagnostic markers for the early detection of tumours. By proteomic approaches, several autoantibodies were proposed as potential markers. We tried in this study, to perform a serological proteome analysis, using various antigenic substrates, including tumours and human liver.

**Methods:**

Sera from patients (n = 13) and healthy donors (n = 10) were probed on immunoblots performed using 2-dimensionally separated proteins from cholangiocarcinoma cell lines (CCLP1 and CCSW1), from the liver of healthy subject and interestingly, from tumour and adjacent non-tumour liver tissues from five patients with cholangiocarcinoma and tested with their corresponding serum. Spots of interest were identified using mass spectrometry and classified according gene ontology analysis.

**Results:**

A comparison of the whole immunoblotting patterns given by cholangiocarcinoma sera against those obtained with normal control sera enabled the definition of 862 spots. Forty-five different proteins were further analysed, corresponding to (1) spots stained with more than four of 13 (30 %) sera tested with the CCLP1 or the CCSW1 cell line and with the normal liver, and (2) to spots immunoreactive with at least two of the five sera probed with their tumour and non-tumour counter-part of cholangiocarcinoma. Immunoreactive proteins with catalytic activity as molecular function were detected at rates of 93 and 64 % in liver from healthy subjects or cholangiocarcinoma non-tumour tissues respectively, compared to 43, 33, 33 % in tumour tissues, or CCSW1 and CCLP1 cell lines. A second pattern was represented by structural proteins with rates of 7 and 7 % in normal liver or non-tumour tissues compared to 14, 33 and 67 % in tumour tissue, CCSW1 or CCLP1 cell lines. Proteins with a binding function were detected at rates of 7 % in non-tumour tissue and 14 % in tumour tissue. Using the extracted tumour tissue, serotransferrin was targeted by all cholangiocarcinoma-related sera.

**Conclusions:**

Immunological patterns depended on the type of antigen substrate used; i.e. tumour versus non tumour specimens. Nevertheless, a combination of multiple autoantibodies tested with the most appropriate substrate might be more sensitive and specific for the diagnosis of cholangiocarcinoma.

**Electronic supplementary material:**

The online version of this article (doi:10.1186/s12967-015-0751-2) contains supplementary material, which is available to authorized users.

## Background

Cholangiocarcinoma (CC) is a primary liver tumour which results from the malignant transformation of epithelial cells in any portion of the bile ducts. The incidence of CC has increased to 18 % of all liver cancers during the past 30–40 years [1]. Its rate of incidence differs as a function of geographical region [2]. The prognosis for CC is poor and treatment options are very limited. This is partly due to its late diagnosis and onset of symptoms; because of this, the early diagnosis of CC using specific biomarkers remains an important challenge. Antigens such as carbohydrate antigens (CA) and carcinoembryonic antigen are released from digestive tract tumour cells, and their detection in the blood would be a valuable tool to diagnose the cancer and monitor its treatment. However, only a few of them appear to be specific biomarkers for CC [3, 4]. Moreover, standard techniques lack sufficient sensitivity, particularly during the early stages of the tumour process, thus hampering their use in routine practice.

Differences in protein expression between normal and tumour tissues have been studied extensively, including in patients with CC [5]. Studies have also targeted secreted proteins that are directly accessible in biological fluids in the context of CC [6, 7].

Other molecules directly accessible in biological fluids are autoantibodies (AAbs), and their presence has been reported in sera from patients suffering from a variety of malignancies [8]. The origin of the immune response in this setting is largely unknown, although it involves mutation, incorrect protein folding, over-expression and also post translational modifications which cause the neo-antigen to be presented to the immune system [8, 9]. Because circulating AAbs are produced in large quantities despite the presence of small amounts of the corresponding antigen, and because of their persistence and stability in sera, they may be of considerable diagnostic value. In the case of primary liver tumours, and more specifically CC, very few studies on AAbs to tumour associated antigen (TAA) have been reported.

We therefore focused in this study on identifying autoantibodies as biomarkers for the diagnosis of CC, using serological proteome analysis (SERPA) which integrates 2D electrophoresis, western blotting and mass spectrometry. A similar approach was recently reported by Rucksaken et al. [10]. By using antigenic preparations from other cholangiocarcinoma cell lines (CCLP1 and CCSW1), from the liver of healthy humans and from tumour and adjacent non-tumour liver tissues from patients with cholangiocarcinoma, we were able to highlight the heterogeneity of autoantigenic patterns reflecting the diversity of the immune response. The classification of autoantibody targets according to gene ontology analysis enabled the definition of two main patterns which depended on the antigenic extract used, i.e., antigens with structural activity if a tumour specimen was used for AAbs screening, and antigens with catalytic activity if non-tumour specimens were used. In this way, it was also possible to identify the most appropriate substrate producing the best sensitivity.

## Methods

**Fig. 1:**
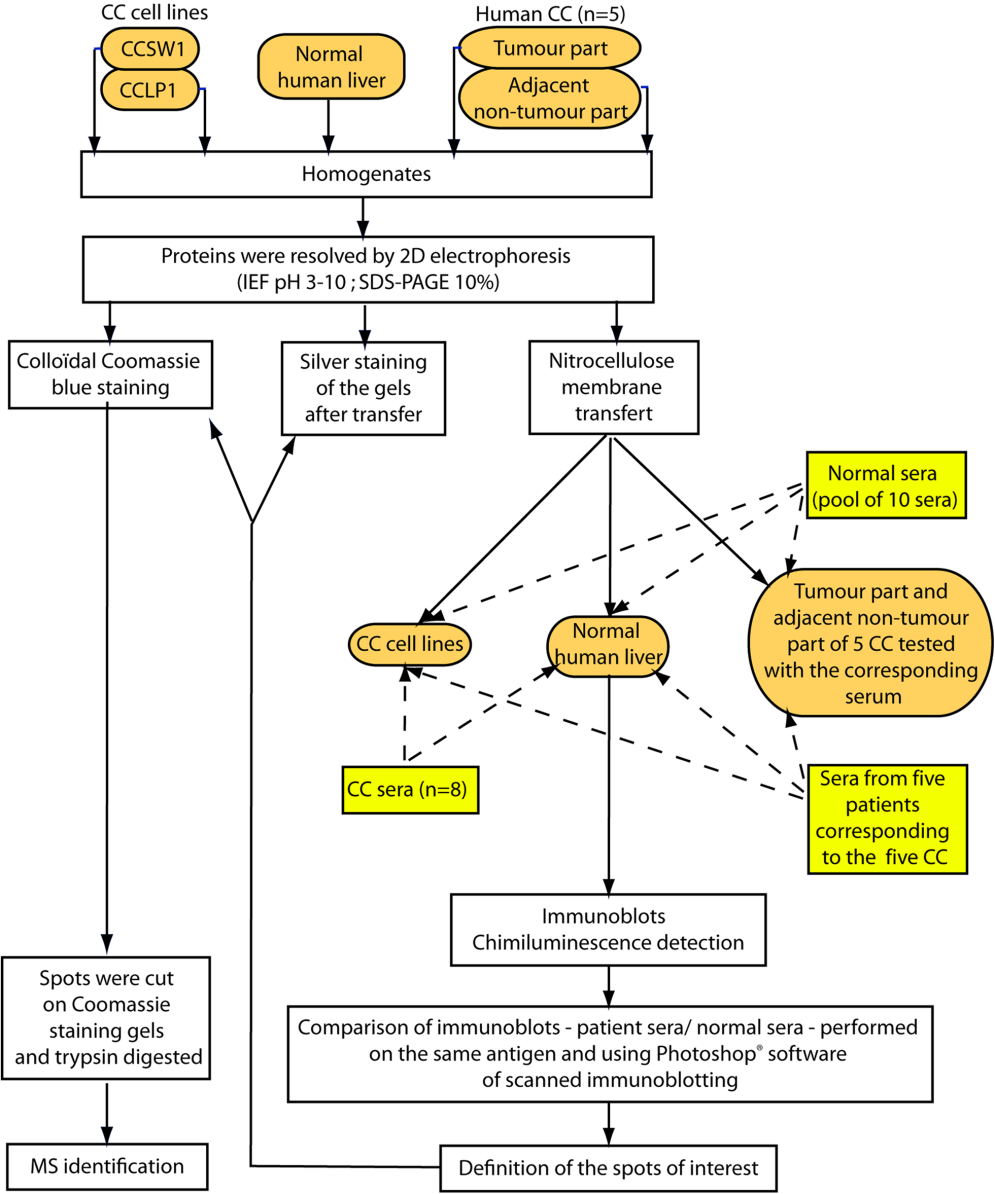
Design of the experiment. *CC* cholangiocarcinoma

The design of the study is summarized in Fig. 1. Briefly, sera from a pool of ten normal subjects, and from eight patients with CC, were tested on two-dimensional immunoblots performed using two CC cell lines and human liver from healthy subjects. In addition, five CC sera were tested on these three substrates and also on immunoblots performed using tumour and non-tumour tissue from the same CC patients. Spots of interest were identified by mass spectrometry (MS), and autoantigens categorised according to the Gene Ontology project before further classification.

### Serum samples and human tissue specimens

All patients gave their informed consent for the collection of blood and tissue samples. Specimens were conserved at −80 °C, with approval of the “Committee of the Biobanque of Centre Hépato-Biliaire”, managed by the “Biological Resource Centre CRB Paris-Sud”. All subjects signed a written informed consent form regarding this analytical study.

Thirteen serum samples from CC patients followed by the Centre Hépato-Biliaire at Hôpital Paul-Brousse, were analysed. All the patients fulfilled the international criteria for the diagnosis of CC. Ten pooled sera from healthy volunteers were used as controls.

The CC tissues and adjacent non-tumour liver tissues used for this study were collected from five CC patients who were being treated surgically in our centre. After resection, the specimens were rinsed thoroughly in ice-cold normal saline and stored at −80 °C. Necrotic tissues were excluded, and pathological examination of the non-tumour liver tissues by an expert (CG) confirmed that they contained no tumour. Normal liver tissue specimens were obtained from patient who had been transplanted for amyloid neuropathy.

All liver tissues were homogenized using a Potter-Elvejhem apparatus, with 10 mM Tris, 50 mM sucrose, 1 mM EDTA and 1 mM phenylmethyl sulphonide fluoride (PMSF). Homogenates were lysed in buffer with 50 mM Tris (pH 7.5), 150 mM NaCl, 1 mM EDTA, 1 % triton (v/v), 0.2 % SDS (w/v) and 1 % (v/v) nuclease mix (GE Healthcare).

### Cell lines

Two human cholangiocarcinoma cell lines, CCSW1 and CCLP1, were obtained from the European Cell Culture Bank, and cells were grown in Dulbecco’s modified Eagle’s medium (DMEM) supplemented with 10 % (v/v) heat inactivated bovine fœtal serum (BFS), 1 % (v/v) minimal essential medium of non-essential amino acids, 1 mmol/L sodium 2-oxopropanoate, and standard concentrations of penicillin plus streptomycin. Whole cell proteins were extracted from the cell lines. Cell lysis was performed with 20 mM Tris (pH 7.5), 150 mM NaCl, 1 % NP40 (Sigma) (v/v), 1× protease inhibitor (Roche, Germany) and 1× phosphatase inhibitor.

### Two-dimensional gel electrophoresis (2-DE) and immunoblotting

Proteins from the lysed homogenates and cell lines were precipitated using the 2-D Clean up kit (GE Healthcare) and the final protein concentration was measured with the 2-D Quant kit (GE Healthcare). Protein samples of 250 μg for future immunotransfer, or 1 mg for future Coomassie blue staining, were mixed with IEF buffer (7.5 M urea, 2.2 M thiourea, 4 % (w/v) CHAPS, 0.6 % (v/v) immobilised pH gradient (IPG) buffer at pH 3–10, 0.8 % (v/v) Destreak^®^ solution (GE Healthcare) and orange G. For each sample, the proteins were applied to an immobiline Dry Strip^®^ (pH range 3–10, 13 cm; GE Healthcare). After overnight rehydration at room temperature, the IEF procedure was performed by applying voltage that was gradually increased to a maximum of 23,000 V/h.

Each IPG strip was then equilibrated with a solution containing 6 M urea, 0.075 M Tris (pH 8.8), 30 % (v/v) glycerol, 2 % (w/v) SDS, 2 % (w/v) DTT and pyronine for 15 min. The strips were equilibrated again by replacing DTT with 5 % (w/v) idoacetamide, for a further 15 min. The IPG strips were applied to 10 % SDS-PAGE for a second dimension protein separation. For subsequent immunoblotting, the proteins were transferred to nitrocellulose membranes and then blocked for 1 h with 50 mL blocking buffer: 5 % (w/v) non-fat powdered milk in TBS-T (Tris Buffer Saline-Tween 20) PH 7.4, (Tris 1 M 2 % (w/v), NaCl 0.8 % (w/v), Tween 0.1 % (v/v). The filters were then probed with sera diluted 1:2000 in TBS-T, and finally incubated with 1:3000 diluted horseradish peroxidase-conjugated anti-human immunoglobulin (BioRad). The proteins were detected by chemiluminescence according to the manufacturer’s instructions (ECL Plus™ Western Blotting Detection kit, GE Healthcare). After transfer, the resulting gels were silver-stained.

The analysis was performed in triplicate. After standard immunoblotting, the patterns produced by CC sera were compared with those given by normal sera using scanning and superimposition by means of Adobe Photoshop^®^ Software. Spots of interest were defined as those which were only stained by CC sera.

The transferred and silver-stained gels and their corresponding immunoblots were also scanned, and after Adobe Photoshop^®^ software analysis, the spots of interest were localised on the silver-stained gels. These spots were then localised together on the corresponding scans of Coomassie blue-stained gels. Immunoreactive spots obtained with at least 30 % of CC sera were then identified using MS.

### Procedures for protein and peptide preparation

The spots of interest were excised manually. Cysteine reduction was performed with 10 mmol/L DTT-100 mmol/L NH_4_HCO_3_ for 45 min at 56 °C, and protein alkylation was carried out with 55 mmol/L iodoacetamide-100 mmol/L NH_4_HCO_3_ for 30 min in the dark at room temperature, the gel pieces being washed successively with 100 mmol/L NH_4_HCO_3_, a 1:1 (by volume) mixture of 100 mmol/L NH_4_HCO_3_ and acetonitrile, and acetonitrile, before being dried again. The gel pieces were then rehydrated for 45 min at 4 °C in a digestion buffer containing 50 mmol/L NH_4_HCO_3_, 5 mmol/L CaCl_2_, and 12.5 mg/L trypsin. Peptides generated through proteolytic digestion were extracted by incubation in 10 g/L formic acid for 15 min, which was followed successively by two extractions with 10 g/L formic acid-acetonitrile (1:1 by volume) and acetonitrile. The extracted peptides were pooled and dried out in a SpeedVac centrifuge before mass spectrometry (MS) analysis.

### Mass spectrometry analysis

LC–MS measurements were obtained using a nano LC system (Ultimate 3000; Dionex) coupled online to a hybrid linear ion trap/Orbitrap^®^ MS (LTQ OrbitrapVelos; Thermo Fisher Scientific, Bremen, Germany). One microlitre of protein digest was injected onto the nano LC system, which contained a C18 trap column (PepMap C18, 300 μmID × 5 mm, 5 μm particle size and 100 Å pore size; Dionex) and a 15 cm long analytical column (Acclaim pepmap RSLC 75 µm × 15 cm, nanoViper C18, 2 µm, 100 Å). The peptides were separated according to the following gradient: 100 % solvent A (0.1 % formic acid in water) for 3 min., 0–55 % solvent B (80 % acetonitrile in water with 0.1 % formic acid) for 25 min., 50–90 % solvent B for 1 min and 90 % solvent B for 5 min. A high resolution full scan MS was obtained from the Orbitrap^®^ (resolution 30,000; AGC 1,000,000), and MS/MS spectra were obtained by CID (collision-induced dissociation) fragmentation, with an isolation window of 3 Da. A data-dependent top 5 (one full MS and 5 MS/MS) was obtained with the dynamic exclusion option switched on. Spots that were reactive with fewer than 30 % of sera were not identified by MS.

### Data analysis

The data were analysed using Discoverer Proteome 1.4 software. The database is a human Swiss-Prot, the mass error for the precursor ions (full MS) being less than 10 ppm. The mass error for ions from the MS/MS spectra is reported to be less than 0.6 Da. Searches for peptide mass are made between 350 and 5000 Da with a time retention ranging from 10 to 50 min. A miss cleavage site is tolerated. Dynamic modification was enabled for N_ter_ acetylation, the oxidation of methionine and histidine, and the carbamidomethylation of amino acids, aspartic acid and glutamic acid. The static carbamidomethyl modification of cysteine was enabled. Peptide identifications are validated by determining false positives using the Target decoy PSM validator. This is high if the false positive rate (FDR or false Discovery Rate) is less than 1 %, low if the FDR is greater than 5 % or average (between 1 and 5 %). Peptide identification Xcorr were calculated by correlating the MS/MS experimental spectrum with the theoretical MS/MS spectrum generated by Proteome Discoverer 1.4 software.

### Detection of anti vimentin and anti actin antibodies by immunofluorescence as a validation technique

The presence of anti-vimentin and anti-actin antibodies was determined using indirect immunofluorescence on monolayers of colchicine-treated Hep2 cells, as described elsewhere [11, 12]. Briefly, Hep2 monolayer cells culture was home-prepared. This culture performed on slide was incubated with colchicine 0.0014 % (w:v) (Sigma) diluted in minimum essential medium Eagle (Eurobio), glutamine supplemented, during 20 h at 37 °C. After three washes with phosphate-buffered saline (PBS), and acetone-fixed, the monolayer cells was incubated with sera at the dilution of 1/40, in PBS, during 30 min. After PBS washing (3×), monolayer cells were revealed using a fluorochrome–labeled, polyclonal antihuman IgG, IgA, IgM antiserum (BioRad Laboratory), 30 min incubated. Vimentin appear to be collapsed into thick perinuclear coils if the tested serum was positive for anti-vimentin antibodies, and a typical pattern of actin cables was strongly stained if serum was positive for anti-actin antibodies.

## Results

### Identification of immunoreactive proteins in cell lines from CC patients

#### CCSW1 cell line

**Fig. 2:**
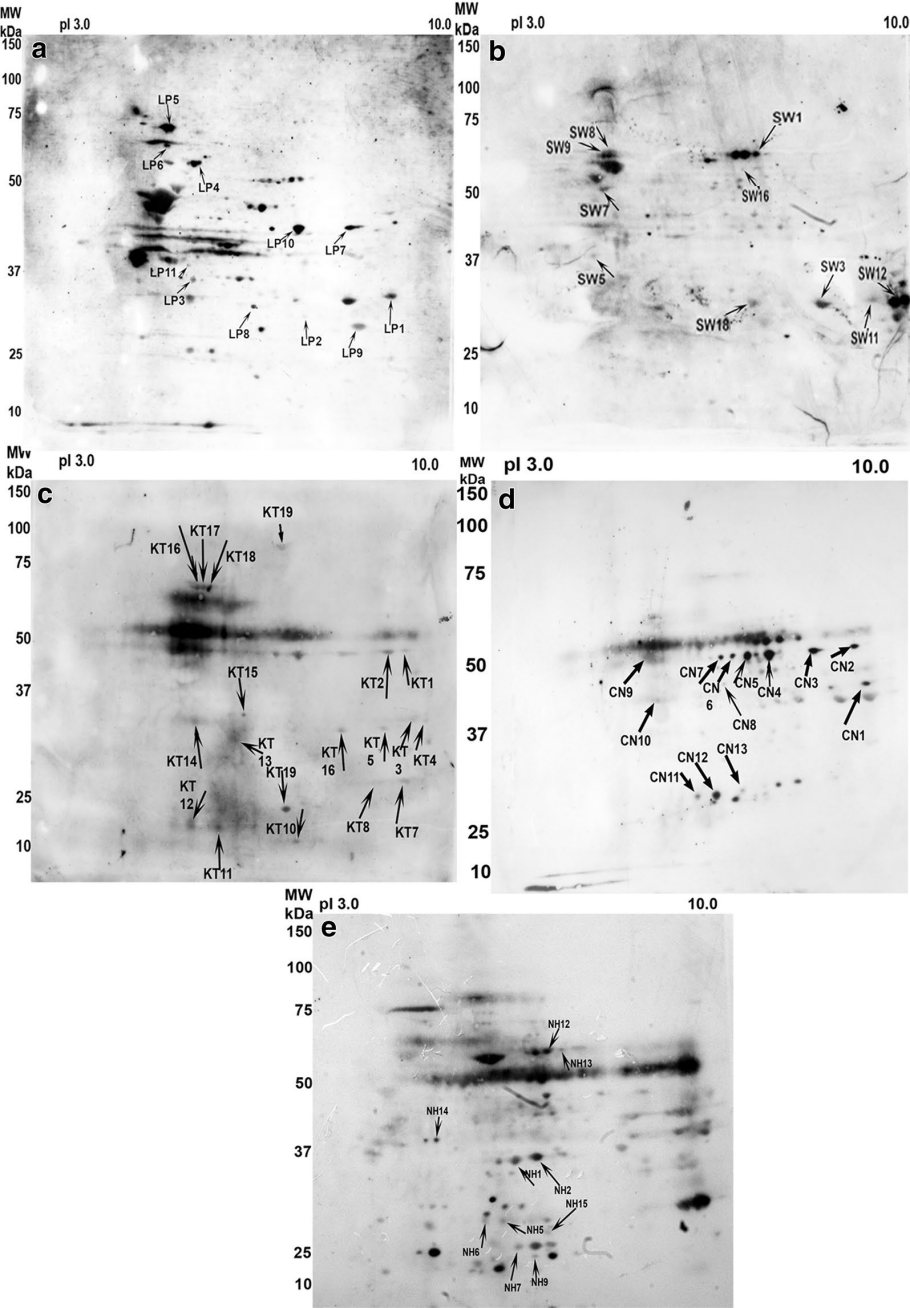
Representative example of the immunoblotting pattern displayed by the same serum tested on the different antigenic extracts. **a** CCLP1 cell line; **b** CCSW1 cell line; **c** tumour part of cholangiocarcinoma; **d** non-tumour part adjacent to the cholangiocarcinoma; **e** normal liver. *Counts with arrows* correspond to the different immunoreactive spots detected on the corresponding Coomassie-stained gel (see Figs. 3, 4, 5, 6, 7) and listed in Additional file 1: Table S1a, b

**Fig. 3:**
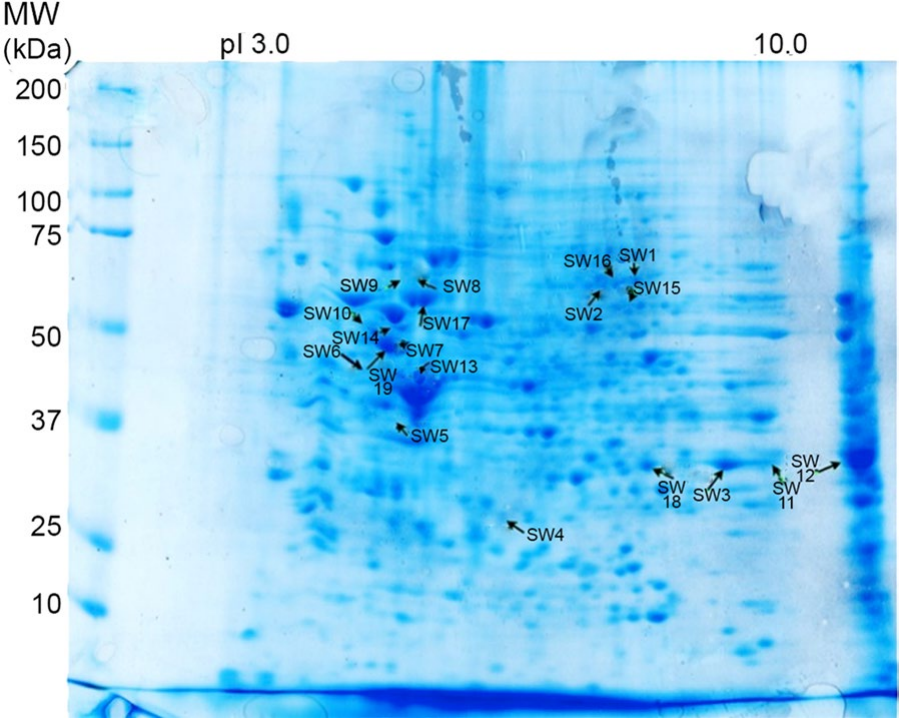
Coomassie-blue stained gel of CCSW1 2-D resolved proteins. Proteins immunoreactive with more than 30 % of the 13 CC sera compared to controls are indicated by *arrows*. These immunoreactive spots are listed in Additional file 1: Table S1. Isoforms of vimentin stained by 100 % of CC sera were located as SW7, SW10, SW14, SW19. Prelamine A/C (SW2, SW16) was recognized by 69 % of sera, annexin A2 (SW3, SW11, SW12, SW18) was a target for 62 % of sera. hnRNP L (stained by 54 % of sera) corresponded to SW1. Dihydrolipoyl dehydrogenase (46 % of sera) corresponded to SW15. Each of the remaining five spots were stained by 31 % of CC sera: actin (SW13), hnRNPC1/C2 (SW5), hnRNP K (SW8, SW9), HSP60 (SW17), and protein phosphatase 1 (SW6)

Using the CCSW1 cell line as the antigen, a comparison of all the immunoblotting patterns given by CC sera against those obtained with normal control sera enabled the definition of a total of 172 spots that were only stained by CC sera. Nineteen of these 172 spots (11 %) were stained with at least one-third (i.e. four sera) of the 13 CC sera and eighteen thereafter identified by MS (Additional file 1: Table S1; Figs. 2 and 3). They corresponded to ten proteins (Table 1): vimentin (four isoforms), which was stained by all 13 of the CC sera, prelamin A/C (two isoforms) recognized by nine out of 13 sera (69 %), annexin A2 (four isoforms) stained by eight sera (62 %), hnRNPL recognised by seven sera (54 %), and dihydrolipoyl dehydrogenase by six sera (46 %). Six spots were immunoreactive with four (31 %) of the 13 CC sera and corresponded to: actin, hnRNP C1/C2, hnRNP K (two isoforms), HSP60, protein phosphatase 1 (Table 1).

**Table 1:** Common immunoreactive proteins identified by CC sera in at least one-third of sera with the different antigenic extracts used

Proteins	Access number	CCSW1	CCLP1	Tumour tissue	Adjacent non-tumour tissue	Normal liver
3-Ketoacyl-CoA thiolase	P42765	NI	NI	NI	2/5 (40 %)	6/13 (46 %)
78 kDa glucose regulated protein	P11021	NI	5/13 (38 %)	NI	I	NI
α-Enolase	P06733	NI	NI	3/5 (60 %)	2/5 (40 %)	NI
β-Enolase	P13929	NI	NI	NI	2/5 (40 %)	NI
Acetyl coA acetyl transferase	P24752	NI	NI	NI	2/5 (40 %)	4/13 (31 %)
Aconitate hydratase	Q99798	NI	NI	NI	NI	5/13 (38 %)
Actin	P60709	4/13 (31 %)	6/13 (46 %)	4/5 (80 %)	I	NI
Aldehyde dehydrogenase	F8W0A9	NI	NI	NI	NI	4/13 (31 %)
Annexin A1	P04083	NI	6/13 (46 %)	I	NI	NI
Annexin A2	P07355	8/13 (62 %)	9/13 (69 %)	2/5 (40 %)	I	NI
Annexin A4	P09525	NI	NI	2/5 (40 %)	NI	NI
Annexin A5	P08758	NI	NI	2/5 (40 %)	NI	NI
ATP bifunctional dihydroxyacetone kinase	Q3LXA3	NI	NI	NI	I	5/13 (38 %)
ATP synthase sub unit α	P25705	NI	NI	3/5 (60 %)	NI	NI
ATP synthase sub unit β	P06576	NI	NI	I	2/5 (40 %)	NI
Carbonic anhydrase 1	P00915	NI	NI	I	NI	4/13 (31 %)
Catalase	P04040	NI	NI	NI	2/5 (40 %)	NI
∆(3,5)-∆(2,4)-dienoyl-CoA isomerase	Q13011	NI	NI	I	I	4/13 (31 %)
∆-1-pyrroline-5-carboxylate dehydrogenase	P30038	NI	NI	NI	NI	4/13 (31 %)
Dihydrolipoyl dehydrogenase	P09622	6/13 (46 %)	NI	I	I	NI
Electron transfer flavoprotein α	P13804	NI	NI	NI	NI	5/13 (38 %)
Epoxyde hydrolase	P07099	NI	NI	NI	2/5 (40 %)	NI
Estradiol 17-β-dehydrogenase 8	Q92506	NI	NI	NI	NI	5/13 (38 %)
Fructose-1.6-biphosphatase 1	P09467	NI	NI	NI	I	5/13 (38 %)
Fructose biphosphate aldolase A	P04075	NI	5/13 (38 %)	NI	NI	NI
Fructose biphosphate aldolase B	P05062	NI	NI	NI	4/5 (80 %)	5/13 (38 %)
Glutathione S-transferase	P09211	NI	4/13 (31 %)	NI	NI	NI
Glyceraldehyde-3-phosphate dehydrogenase	E7EUT4	NI	NI	I	I	7/13 (54 %)
hnRNP C1/C2	G3V4C1	4/13 (31 %)	NI	NI	NI	NI
hnRNP K	P61978	4/13 (31 %)	NI	NI	NI	NI
hnRNP L	P14866	7/13 (54 %)	NI	NI	NI	NI
HSP1 β1	P04792	NI	7/13 (54 %)	I	NI	NI
HSP 60	P10809	4/13 (31 %)	NI	NI	3/5 (60 %)	NI
Lamin B2	Q03252	NI	5/13 (38 %)	NI	NI	NI
Liver arginase (arginase 1)	P05089	NI	NI	NI	2/5 (40 %)	7/13 (54 %)
Liver carboxyl-esterase 1	E9PAU8	NI	NI	NI	2/5 (40 %)	NI
Prelamine A/C	P02545 P02545-2	9/13 (69 %)	NI	I	3/5 (60 %)	4/13 (31 %)
Proteasome su α2	P25787 G3V295	NI	NI	2/5 (40 %)	NI	NI
Protein phosphatase 1	Q15435	4/13 (31 %)	NI	NI	NI	NI
Retinal dehydrogenase 1	P00352	NI	4/13 (31 %)	I	2/5 (40 %)	NI
Serine hydroxymethyltransferase	P34896	NI	5/13 (38 %)	NI	NI	NI
Serotransferrin	P02787	NI	NI	5/5 (100 %)	I	NI
Serum albumin	P02768	NI	NI	2/5 (40 %)	3/5 (60 %)	NI
S-methyl-5′ thioadenosine phosphorylase	Q13126	NI	NI	NI	NI	5/13 (38 %)
Vimentin	P08670	13/13 (100 %)	4/13 (31 %)	I	NI	NI

*NI* not identified, *I* recognized by less than 31 % of CC sera. Access numbers are from the Swiss-Prot database

#### CCLP1 cell line

**Fig. 4:**
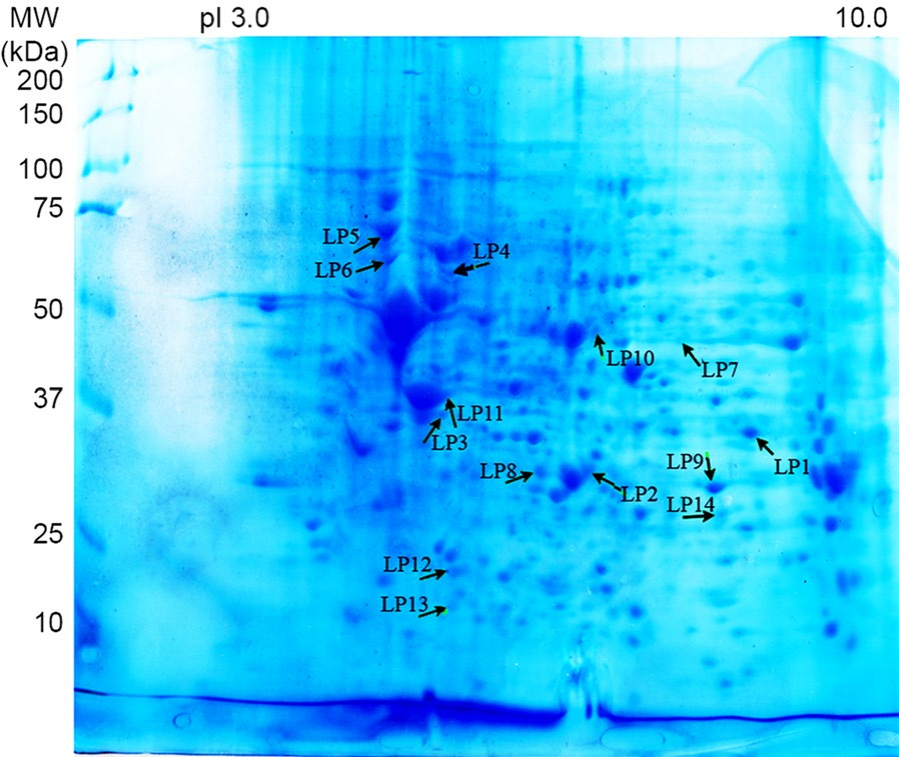
Coomassie-blue stained gel of CCLP1 2-D resolved proteins. Proteins immunoreactive with more than 30 % of the 13 CC sera are highlighted by *arrows*. These immunoreactive spots are listed in Additional file 1: Table S1. Isoforms of annexin A2 were recognized by 69 % of CC sera and corresponded to spots LP9 and LP14. HSP-β1 (54 % of sera) corresponded to LP12. Isoforms of annexin A1 and actin were recognized by 46 % of CC sera and corresponding spots were LP2 and LP8 (for annexin A1) and LP3 and LP11 (for actin). Fructose-biphosphate aldolase A (LP1), lamin-B2 (LP4), 78 kDa glucose-regulated protein (LP5) and isoform 2 of serine hydroymethyltransferase (LP7) were identified by 38 % of CC sera. Each of the remaining three spots were stained by only four (31 %) different sera: glutathione S-transferase (LP13), retinal dehydrogenase 1 (LP10) and vimentin (LP6)

Concerning the CCLP1 cell line, 189 spots were stained by CC sera only, but only 14 spots corresponding to eleven identified proteins were immunoreactive with more than four (30 %) out of 13 sera (Additional file 1: Table S1; Table 1; Figs. 2, 4). Annexin A2 (two isoforms) reacted with nine of the 13 CC sera (69 %), and heat shock protein (HSP) β-1 with seven sera (54 %). Actin (two isoforms) and annexin A1 (two isoforms) were recognised by six (46 %) of the 13 CC sera. Fructose-bisphosphate aldolase A, lamin-B2, 78 kDa glucose-regulated protein (GRP78), and isoform 2 of serine hydroxymethyltransferase were stained by five (38 %) of the 13 CC tested sera, whereas gluthathione S-transferase, retinal dehydrogenase and vimentin were only recognised by four (31 %) of the CC sera.

### Immunoreactive protein spots in human liver from healthy subject

**Fig. 5:**
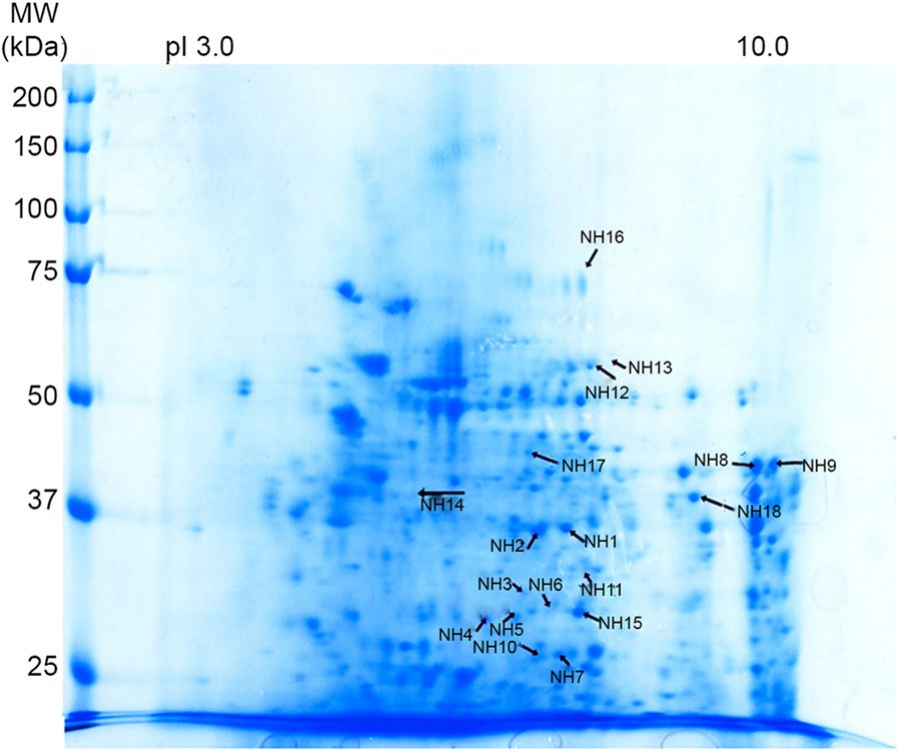
Coomassie-blue stained gel of normal liver 2-D resolved proteins. Proteins immunoreactive with more than 30 % of the 13 CC sera are indicated by *arrows* and listed in Additional file 2: Table S2. Liver arginase 1 corresponding to arrows NH1 and NH2 and glyceraldehyde-3-phosphate dehydrogenase (NH6, NH11) were recognized by 54 % of 13 CC sera, 3 ketoacyl-COA thiolase corresponded to arrows NH8 and NH9 (46 % of CC sera). Aconitate hydratase (NH16), bifunctional ATP-dependant dihydroxyacetone kinase (NH13), electron transfer-flavoprotein α (NH15), estradiol 17-β-dehydrogenase 8 (NH3), fructose-1.6 biphosphatase 1 and fructose-biphosphate aldolase B (both identified in the same spot NH10), S-methyl-5′ thioadenosine phosphorylase (NH7), were each recognized by 38 % of the CC sera. Proteins recognized by 31 % of the sera were: acetyl CoA acetyl transferase mitochondrial (NH18), aldhehyde dehydrogenase (NH14), carbonic anhydrase 1 (NH5), Δ(3,5) Δ(2,4) dienoyl Coa isomerase (NH4), Δ-1-pyrroline-5-carboxylate dehydrogenase (NH12) and prelamine A/C (NH17)

By comparing the immunoblots of normal liver specimens, 270 spots were specifically stained by CC sera, of which 18 were recognized by more than four (31 %) out of 13 sera and identified by MS (Additional file 2: Table S2; Figs. 2, 5). They corresponded to 16 proteins resulting from the existence of isoforms (Table 1). Liver arginase 1 (two isoforms) and glyceraldehyde-3-phosphate dehydrogenase (two isoforms) each reacted with seven (54 %) of the 13 CC sera, and 3-ketoacyl-CoA thiolase (two isoforms) with six (46 %) sera. Seven proteins corresponding to six spots were stained by five (38 %) of the 13 CC sera: aconitate hydratase, bifunctional ATP-dependent dihydroxyacetone kinase, electron transfer-flavoprotein α, estradiol 17 β dehydrogenase 8, fructose-1.6 biphosphatase 1 and fructose-biphosphate aldolase B (both identified in the same spot with a high probability), and S-methyl-5′ thioadenosine phosphorylase, The remaining six spots were stained by four (31 %) of the 13 CC sera: acetyl coA acetyl transferase mitochondrial, aldhehyde dehydrogenase, carbonic anhydrase 1, Δ(3,5) Δ(2,4) dienoyl CoA isomerase, Δ1-pyrroline-5-carboxylate dehydrogenase and prelamine A/C.

### Reactivity patterns of immunoreactive spots in human tumour and adjacent non-tumour tissues

#### Tumour part of the choliangiocarcinoma

**Fig. 6:**
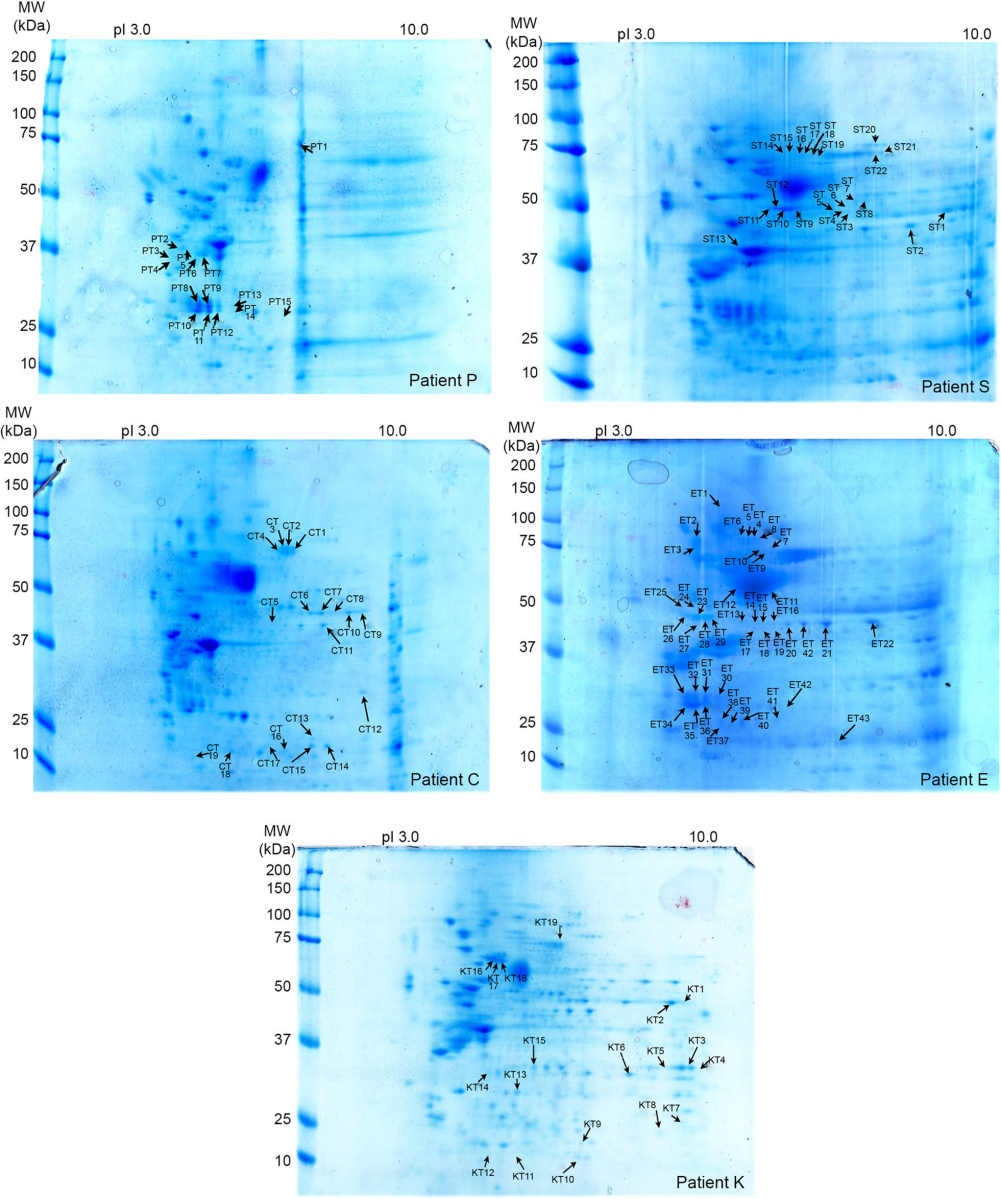
Coomassie-blue stained gels of 2D-resolved proteins from five tumour-affected CC livers. Each tumour extract was tested with serum from the corresponding patient (C, E, K, P and S). *Arrows* (CT, ET, KT, PT and ST) indicate the immunoreactive proteins that were only stained by CC sera. They are listed in Additional file 1: Table S1. Widespread immunoreactive spots were noted, depending on the CC serum tested. Of the 39 different proteins recognized by the CC sera, only nine were reactive with more than one-third of sera (Table 1). Serotransferrin was identified by 100 % of the five CC sera, actin by four (80 %), and ATP synthase subunit-α and α-enolase were each stained by three (60 %) of the CC sera. Some proteins were immunoreactive with two CC sera (40 %): annexin A2, A4 and A5, proteosome subunit-α type-2 and serum albumin

Concerning the five tumour antigen extracts tested by immunoblotting with the corresponding patient’s serum and then compared against the pattern obtained with control sera, widespread immunoreactive spots (n = 118) were noted depending on the CC serum tested. Thirty-nine proteins were identified by MS (Additional file 1: Table S1; Figs. 2, 6), but only nine were reactive with more than one-third of the sera (Table I). Serotransferrin was identified by 100 % of the five CC sera. Actin was stained by four (80 %) of the five sera tested, and ATP synthase subunit-α and α-enolase were each stained by three (60 %) of the five CC sera. Some proteins were immunoreactive with two (40 %) CC sera: annexin A2, A4 and A5, proteasome subunit-α type-2 and serum albumin.

#### Non-tumour tissue adjacent to the cholangioacarcinoma

**Fig. 7:**
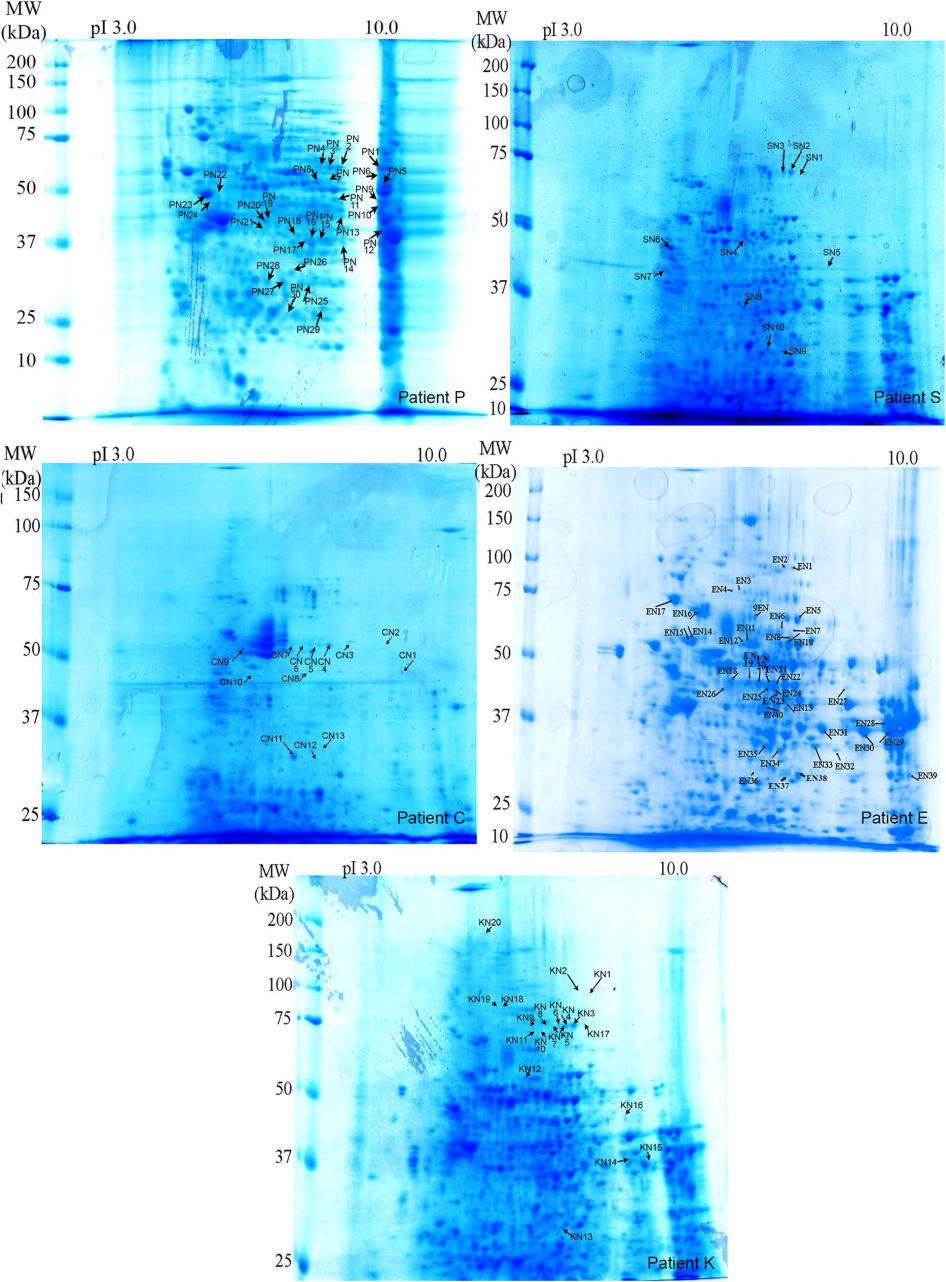
Coomassie-blue stained gels of 2D-resolved proteins from five non-tumour tissues adjacent to the CC livers. Each extract was tested with the serum of the corresponding patient (C, E, K, P and S). *Arrows* (CN, EN, KN, PN and SN) indicate the immunoreactive proteins stained only by CC sera. They are listed in Additional file 2: Table S2. As for their non-tumour counterparts, a widespread immunopattern was noted. Fifty-eight different proteins were identified. Fourteen proteins were selectively stained by more than three (30 %) of the CC sera (Table 1). Fructose-bisphosphate aldolase B was identified by four (80 %) of the patient sera, HSP60, prelamine A/C and serum albumin by three (60 %). Ten proteins were targets for two (40 %) of the CC sera: 3-ketoacyl-CoA thiolase, α-enolase, β-enolase, acetyl-CoA acetyltransferase, ATP synthase subunit β, catalase, epoxyde hydrolase, liver arginase, liver carboxylesterase 1 and retinal dehydrogenase

As for their non-tumour counterparts, a widespread immunopattern was noted. A total of 113 spots were stained, corresponding to 58 identified proteins, indicating the existence of isoforms (Additional file 2: Table S2; Figs. 2, 7). Fourteen proteins were selectively stained by more than three (30 %) of the five CC sera (Table 1). Fructose-bisphosphate aldolase B was identified by four (80 %) of the five patient sera. While HSP60, prelamine A/C and serum albumin were reactive with three (60 %) of the CC sera. Ten proteins were targets for two (40 %) of the five CC sera: 3-ketoacyl-CoA thiolase, α-enolase, β-enolase, acetyl-CoA acetyltransferase, ATP synthase subunit β, catalase, epoxyde hydrolase, liver arginase, liver carboxylesterase 1 and retinal dehydrogenase.

### Gene ontology analysis

To obtain a comprehensive view of these different immunoreactive proteins, antigens that were recognised by more than 30 % of the CC sera were grouped according to their elemental activity, the molecular function ontology (Table 2), and at the cellular level, to their biological process ontology (Table 3). At the cellular level, recognized antigens were also grouped according to their protein class (Additional file 3: Table S3) and to their molecular pathway (Additional file 4: Table S4), as defined by the Panther classification (http://www.pantherdb.org).

**Table 2:** Gene ontology distribution of proteins recognized by CC sera, according to molecular functions

	CCSW1	CCLP1	Tumour part	Adjacent non-tumour part	Normal liver
Catalytic activity	33.3 %	33.3 %	42.9 %	64.3 %	92.9 %
Structural molecule	33.3 %	66.7 %	14.3 %	7.1 %	7.1 %
Binding	33.3 %	–	14.3 %	7.1 %	–
Receptor activity	–	–	14.3 %	7.1 %	–
Antioxidant activity	–	–	–	7.1 %	–
Transporter activity	–	–	14.3 %	7.1 %	–

**Table 3:** Gene ontology distribution of proteins recognized by CC sera, according to biological process

	CCSW1	CCLP1	Tumour part	Adjacent non-tumour part	Normal liver
Metabolic process	31.6 %	26.1 %	42.9 %	66.7 %	81.3 %
Cellular process	21.1 %	17.4 %	7.1 %	6.7 %	6.3 %
Developmental process	21.1 %	17.4 %	14.3 %	6.7 %	6.3 %
Cellular component organization or biogenesis	15.8 %	17.4 %	7.1 %	6.7 %	6.3 %
Localization	10.5 %	4.3 %	28.6 %	13.3 %	–
Immune system process	–	4.3 %	–	–	–
Multicellular organism process	–	4.3 %	–	–	–
Response to stimulus	–	8.7 %	–	–	–

Non-tumour specimens, i.e., normal liver and non-tumour tissues adjacent to the CC, contained a high percentage of auto-antigenic proteins categorized as catalytic activity as a molecular function (92.9 and 64.3 % respectively) (Table 2) when compared to tumour specimens or CCSW1 or CCLP1 cell lines (42.9, 33.3 and 33.3 %, respectively), thus explaining the predominance of auto-antigens with metabolic process as biological process recognized in normal liver (81.3 %) and in non-tumour tissues adjacent to the CC (66.7 %) (Table 3) compared to CC tumour tissues (42.9 %) and also in CCSW1 and CCLP1 cell lines (31.6 and 26.1 %). Proteins classified as oxydoreductase or transferase as a molecular pathway displayed the same distribution (Additional file 3: Table S3). They constituted a large share of the antigens recognised in normal liver (at rates of 28.6 and 23.8 % respectively). Oxydoreductase and transferase were less or not recognised in other antigenic substrates. Findings were similar in the protein pathway group (Additional file 4: Table S4) in which enzymes for fructose galactose metabolism and glycolysis were detected in normal liver at rates of 12.5 and 25.0 %, respectively, and in non-tumour liver specimens at 20.0 and 40.0 %. Lower rates were found in tumour specimens (0 and 9.1 %, respectively), in the CCSW1 cell line (0 and 0 %) and in the CCLP1 cell line (6.3 and 6.3 %). It is also interesting to note that enzymes involved in ATP synthesis (Additional file 4: Table S4) were preferentially recognised in non-tumour tissues adjacent to the CC (20.0 %) compared to CC tumour tissues (9.1 %).

As for molecules with structural activity as a molecular function (Table 2), they were preferentially recognised in the CCSW1 cell line (33.3 %), the CCLP1 cell line (66.7 %) and to a lesser extent in tumour specimens (14.3 %). The rates were lower if the antigens were from non-tumour tissues adjacent to the CC (7.1 %) or from normal liver (7.1 %). They corresponded to cytoskeletal protein or structural protein as protein classes (Additional file 3: Table S3) majority found in CC cell lines.

In addition, recognised proteins belonged to the transfer/carrier class (Additional file 3: Table S3) were predominant in cancer tissue. They represented 20.0 % of the AAbs targets tested on CC tumour tissues, compared to 6.3 % on non-tumour tissues adjacent to the CC.

At least, several members of the annexin family were targeted by 40–69 % of CC sera, but only if tumour specimens were used for screening (Table 1). They were involved in various biological processes, such as a cellular process for all isoforms, as well as a metabolic process for the A1 isoform or a developmental process for the A2 isoform (Table 3), and as a molecular pathway for the A5 isoform, the gonadotrophin releasing hormone receptor (Additional file 4: Table S4).

### Validation of several antigenic targets

Among the various AAbs found, we chose to use a fluorescence technique which, unlike SDS-PAGE, can offer access to conformational epitopes and hence detect on colchicine-treated Hep2 cells, anti-vimentin antibodies, and consequently anti-actin antibodies. A typical immunofluorescence pattern reflecting anti-vimentin was observed with eight (61 %) of the 13 CC. Among the six sera reacting with actin by immunoblotting, three produced a typical pattern of actin cable (Additional file 5: Figure S1).

## Discussion

This study highlights the heterogeneity of autoantigen patterns that reflect the diversity of the immune response as a function of the serum tested, the different fractions used; thus, as previously, it has underlined the specific nature of the immune response in the setting of cancer [5]. Our data are in accordance with the recent report by Rucksaken [10] which demonstrated that CC sera contain AAbs reacting with enolase and heterogeneous nuclear ribonucleoprotein L. However, we were not able to detect AAbs to ribonuclease/angiotensin inhibitor 1 or to heat shock protein 70, as reported by the same authors. It was nevertheless not surprising to observe different immunoreactive patterns being displayed by CC sera on the different antigenic extracts used, probably due to the specific nature of the cancer cells involved or the technique employed. It is postulated that autoantibodies in cancer are induced by a breakdown in self-tolerance resulting from over-expression, mutations, changes to post-translational modifications or the truncation of proteins in a cancer cell [7]. One hallmark of cancer is genome instability, which can differ from one cell to another and of course from normal cells to cancer cells [8]. In terms of protein expression and modification, choliangocarcinoma cell lines differed from the five tumour extracts, which in turn differed from each other and also from non-tumour specimens. It follows that a protein may be abnormally autoantigenic regarding induction of the autoimmune process, and not be present in the substrate used for AAb screening.

Secondly, the hepatocytes and cholangiocytes present in the liver and cholangiocarcinoma cell lines have different metabolic activities or cellular specializations with differencies on the level of expression of antigenic targets.

Thirdly, the 2D electrophoresis technique used during our study involved a whole homogenate, implying a bias towards abundant proteins.

Added to the previous considerations, some of the proteins resolved were found in sufficient quantities to be immunoreactive when transferred to a nitrocellulose membrane, whereas others were not; for example, nuclear proteins other than histones.

Taken together, these considerations may explain the variability of the immunoblotting patterns we noted and which differed somewhat from the results obtained by Rucksaken [10].

The Gene Ontology classification of autoantigenic targets as a function of their origin revealed two patterns of molecular function: catalytic activity or structural activity (Table 2).

Identified proteins displaying catalytic activity were mainly found when liver was used as antigens, including normal liver or non-tumour tissues adjacent to the CC, and to a lesser extent to tumour-affected part of the liver. Hepatocytes are the principal site for carbohydrate metabolism, and three of the main targets we found (identified by at least 40 % of CC sera with the most appropriate antigenic extracts) were enzymes implicated more specifically in glycolysis and fructose-galactose metabolism: i.e., alpha-enolase, fructose biphosphate aldolase and glyceraldehyde3-phosphate dehydrogenase. Interestingly, these three enzymes were found in the secretome of a cholangiocarcinoma cell line HuCCA1 [6]. Furthermore, enolase and glyceraldehyde3-phosphate dehydrogenase were reported as being over-expressed through study of the HuCCA1 proteome [5]. Even more interesting, and mentioned above, was the report of enolase as an antigenic target for CC sera, using substrates other than those we used [10]. More generally, by probing a protein array with numerous sera from patients with a variety of cancers, increased reactivity to glycolytic enzymes has been reported [13].

However, these three targets were not all specific to CC sera and have been reported (sometimes at a high frequency) in liver diseases, including hepatocellular carcinoma [14, 15], as well as glyceraldehyde3-phosphate dehydrogenase in melanoma [16], enolase in some autoimmune diseases and infections [17–19] and fructose biphosphate aldolase in a case of drug hepatotoxicity [20].

Concerning other immunoreactive targets identified by fewer CC sera and having catalytic activity, i.e., liver arginase and ATP synthase sub-unit β, they have been reported in a variety of settings including autoimmune diseases [21], Alzheimer’s disease [22], coeliac disease [23], idiopathic nephrotic syndrome [24] and also in 11 % of hepatocellular carcinoma patients [25]. However, to our knowledge, ATP synthase sub-unit β has never previously been reported as an auto-antigen. Interestingly, ATP synthase is also located at the cell surface and may contribute to the development of an acidic micro-environment in tumour tissues [26]. Its surface location allows it to gain access to the immune system, and it has been reported as the target of a subset of T-gamma-delta lymphocytes [27].

In terms of antigenic targets with structural activity, they were essentially found when CC cell lines or tumour liver specimens were used as antigens. Three of them were identified by more than 50 % of CC sera on the most appropriate substrate, i.e., vimentin, prelamine A/C and actin. Both actin and members of the intermediate filament family have been demonstrated to be strongly implicated in tumorogenesis [28–30]. But AAbs to these proteins lack specificity as potential biomarkers, because they may be detected in various autoimmune disorders [31], in liver diseases [32], and in hepatocellular or digestive cancer [32, 33]. However, to our knowledge, AAbs to actin have never been reported in the context of liver carcinoma. The presence of AAbs to vimentin and actin was confirmed using a fluorescence technique that enabled access to a conformational epitope, whereas serological proteome analysis generates denatured antigens. Interestingly, actin has been reported to be found in the secretome of HuCCA CC cell lines [6], and its presence in a bodily fluid may cause a loss of immune tolerance. In this study of Srisomsap [6], annexin was also identified in the secretome. We also found various isoforms of annexin, A1, A2, A4 and A5 as the principal antigenic targets for CC sera if they were tested on a tumour substrate (Table 1). Annexin is involved in many cellular processes [34], and is implicated in the genesis of numerous diseases. Annexin A1 was recently reported to be highly expressed in CC, but not in hepatocellular carcinoma [35]. The over-expression or post-translational modification of annexin A2 has been reported in various cancers, such as colorectal, oral and lung cancers [36–40]. However, AAbs to annexin A2 have also been reported in the context of anti-phospholipid syndrome, sometimes in association with cancer [41, 42].

One final and interesting observation was the presence of proteins categorised as transfer/carrier proteins and accounting for 20 % of autoantigenic targets in the tumour tissues, compared to 6.3 % in the non-tumour tissues adjacent to the CC (Additional file 3: Table S3). These proteins included serotranferrin, which was recognised by 100 % of CC sera. Serotansferrin carries iron from its absorption sites and delivers the metal to cells [43], and may also contribute to stimulating cell proliferation [44]. It was very exciting to note that once again, serotransferrin was identified in the secretome of CC cell lines [6]. Until now, anti-serotransferrin auto-antibodies had been found in 30 % of sera from patients with hepatocellular carcinoma and at a lower rate of 5 % in the context of liver cirrhosis and chronic hepatitis [15].

## Conclusion

In order to be used as biomarkers, AAbs need to be both highly sensitive and highly specific. However, most of the AAbs detected during the present study had previously been reported not only in cancers, but also in the context of autoimmune disorders. We therefore cannot conclude that AAb alone could be considered as a biomarker, in agreement with a previous report [10]. Nevertheless, a combination of several AAbs tested on a panel of a significant number of patients, and using the most appropriate substrate defined during this study, might be able to identify the best biomarkers for CC.

## Additional files

10.1186/s12967-015-0751-2 Identification of all immunoreactive proteins in the CCLP1 and CCSW1 cell lines and in the five tumour-affected livers. Spots with the SW abbreviation correspond to those indicated in Fig. 3 and stained by more than one-third cholangiocarcinoma sera with the CCSW1 cell line. Those with the LP abbreviation are those indicated in Fig. 4 with the CCLP1 cell line, stained by more than one-third of sera. Spots with the CT, ET, KT, PT, or ST abbreviations correspond to those indicated on the five gels in Fig. 6 and stained by the patient’s serum reacting with its own tumour liver proteins.
10.1186/s12967-015-0751-2 Identification of immunoreactive proteins in normal liver and in the five non-tumour counterparts adjacent to the cholangiocarcinoma. Spots with the NH abbreviation correspond to those indicated in Fig. 5 and stained by more than one-third of cholangiocarcinoma sera on normal liver. Spots with the CN. EN. KN. PN. or SN abbreviations, correspond to those indicated on the five gels in Fig. 7 and stained by the patient’s serum reacting with its own non-tumour liver proteins adjacent to the cholangiocarcinoma.
10.1186/s12967-015-0751-2 Gene Ontology distribution of proteins recognized by CC sera, according to the protein class.
10.1186/s12967-015-0751-2 Gene Ontology distribution of proteins recognized by CC sera, according to the pathway involved.
10.1186/s12967-015-0751-2 Anti-actin and anti-vimentin evaluation using immunofluorescence on colchicine-treated Hep2 cells. Sera positive by MS for anti-actin or anti-vimentin autoantibodies were tested by indirect immunofluorescence; (A) typical pattern of actin-cable strongly stained by anti-actin antibody; (B) typical pattern given by anti-vimentin antibody, vimentin colchicine-treated collapses into perinuclear coils.

## Abbreviations

2-D: two-dimensional
AAb: autoantibody
CC: cholangiocarcinoma
IEF: isoelectrofocalisation
MS: mass spectrometry
TAA: tumor associated antigen
HCC: hepatocellular carcinoma
