# Supplementary material for: Autoantibody signatures defined by serological proteome analysis in sera from patients with cholangiocarcinoma
Source: J Transl Med. 2016 Jan 16;14:17. doi: 10.1186/s12967-015-0751-2 (PMC4715332; doi:10.1186/s12967-015-0751-2)
Supplement: Supplementary file 1 — 10.1186/s12967-015-0751-2 Identification of all immunoreactive proteins in the CCLP1 and CCSW1 cell lines and in the five tumour-affected livers. Spots with the SW abbreviation correspond to those indicated in Fig. 3 and stained by more than one-third cholangiocarcinoma sera with the CCSW1 cell line. Those with the LP abbreviation are those indicated in Fig. 4 with the CCLP1 cell line, stained by more than one-third of sera. Spots with the CT, ET, KT, PT, or ST abbreviations correspond to those indicated on the five gels in Fig. 6 and stained by the patient’s serum reacting with its own tumour liver proteins. [file 12967_2015_751_MOESM1_ESM.doc]

**Supplementary file 1: Table S1**. Identification of all immunoreactive proteins in the CCLP1 and CCSW1 cell lines and in the five tumour-affected livers. Spots with the SW abbreviation correspond to those indicated in Figure 3 and stained by more than one-third cholangiocarcinoma sera with the CCSW1 cell line. Those with the LP abbreviation are those indicated in Figure 4 with the CCLP1 cell line, stained by more than one-third of sera. Spots with the CT, ET, KT, PT, or ST abbreviations correspond to those indicated on the five gels in Figure 5 and stained by the patient’s serum reacting with its own tumour liver proteins. Access numbers are from the Swiss-Prot database.

| **Protein Identification** | **Access number** | **Spot No** | **Number of reactive sera** | **Sequence coverage %** | **Score** | **Number of peptides matched** | **Molecular Weight (MW)** | | **Isoelectric Point (pI)** | |
| --- | --- | --- | --- | --- | --- | --- | --- | --- | --- | --- |
|  |  |  |  |  |  |  | **Theoretical** | **Observed** | **Theoretical** | **Observed** |
| 60 kDa heat shock protein, mitochondrial [CH60_HUMAN] | P10809 | SW17 | 4 | 90,92 | 1768,57 | 62 | 61,0 | 58 | 5,87 | 5,3 |
| 78 kDa glucose-regulated protein [GRP78_HUMAN] | P11021 | LP5 | 5 | 74,46 | 1713,01 | 61 | 72,3 | 70 | 5,16 | 4,8 |
| Actin, cytoplasmic 1 [ACTB_HUMAN] | P60709 | LP3,LP11  SW13  ST13  ET30-33,37,38*-41  PT6,7  KT14 | 6  4  1  1  1  1 | 76,00  72,53  64,27  67,47  57,07  36,53 | 800,82  150,58  238,65  91,33  136,89  25,61 | 10  8  7  8  5  4 | 41,7 | 37  44  40  27-30  36  32 | 5,48 | 5,4  5,1  4,8  4.8-5,8  5,2  5,6 |
| Actin-related protein 3 [ARP3_HUMAN] | P61158 | ET13 | 1 | 61,48 | 88,63 | 15 | 47,3 | 43 | 5,88 | 6.0 |
| Alpha-1-antitrypsin [A1AT_HUMAN] | P01009 | ET24-28 | 1 | 48,33 | 145,89 | 12 | 46,7 | 45 | 5,59 | 4,7-5.0 |
| Alpha-enolase [ENOA_HUMAN] | P06733 | ST2  ET14-17,20-22,42  CT11 | 1  1  1 | 86,18  93,55  73,73 | 379,36  610,49  395,74 | 33  11  18 | 47,1 | 44  43  41 | 7,39 | 8,3  6.0-7,9  7,5 |
| Alpha-soluble NSF attachment protein [SNAA_HUMAN] | P54920 | PT9 | 1 | 71,86 | 66,73 | 10 | 33,2 | 28 | 5,36 | 5,5 |
| Annexin A1 [ANXA1_HUMAN] | P04083 | LP2,LP8  KT15 | 6  1 | 95,09  79,48 | 827,78  307,16 | 33  22 | 38,7 | 30  33 | 7,02 | 6.2-6.7  6,5 |
| Annexin A2  [ANXA2_HUMAN] | P07355 | LP9,LP14  SW3 ,11,12,18  CT12  KT6 | 9  8  1  1 | 94,99  92,92  58,70  79,35 | 1728,59  1562,88  102,63  528,95 | 44  42  12  32 | 38,6 | 27-29  33  28  32 | 7,75 | 8,2  8,5-10.0  8,6  8,4 |
| Annexin A4 [ANXA4_HUMAN] | P09525 | PT14  KT13 | 1  1 | 77,74  85,89 | 403,47  482,51 | 20  31 | 35,9 | 26,5  26 | 6,13 | 5,9  6,2 |
| Annexin A5 [ANXA5_HUMAN] | P08785 | ET34-36  PT8,10,11,12 | 1  1 | 94,69  91,88 | 554,68  763,63 | 34  26 | 35,9 | 30  27 | 5,05 | 4,9  5.1-5,3 |
| Apolipoprotein [APOE_HUMAN] | P02649 | PT13 | 1 | 66,88 | 119,35 | 13 | 36,1 | 28 | 5,73 | 5,9 |
| ATP synthase subunit alpha, mitochondrial [ATPA_HUMAN] | P25705 | ST1  CT8,9,10  KT1,2 | 1  1  1 | 70,71  61,30  65,64 | 266,81  229,67  652,88 | 33  19  33 | 59,7 | 49  43  47 | 9,13 | 9.0  7.9-8,1  9,2 |
| ATP synthase subunit beta, mitochondrial [ATPB_HUMAN] | P06576 | ET23,29 | 1 | 77,69 |  | 24 | 56,5 | 45 | 5,40 | 5,4 |
| Carbonic anhydrase 1 [CAH1_HUMAN] | P00915 | CT13,14 | 1 | 61,69 | 140,03 | 8 | 28,9 | 22 | 7,12 | 7.2-7,4 |
| Cathepsin D OS=Homo sapiens [CATD_HUMAN] | P07339 | ET38* | 1 | 47,33 | 120,81 | 11 | 44,5 | 27 | 6,54 | 5,6 |
| Delta(3,5)-Delta(2,4)-dienoyl-CoA isomerase, mitochondrial [ECH1_HUMAN] | Q13011 | PT15 | 1 | 79,57 | 348,66 | 17 | 35,8 | 26 | 8,00 | 7.0 |
| Dihydrolipoyl dehydrogenase, mitochondrial [DLDH_HUMAN] | P09622 | SW15  ST6-8 | 6  1 | 65,42  37,33 | 387,65  79,42 | 22  12 | 54,1 | 57  50 | 7,85 | 7,4  7,4 |
| Electron transfer flavoprotein subunit beta [ETFB_HUMAN] | P38117 | KT7 | 1 | 68,63 | 97,83 | 11 | 27,8 | 26 | 8,10 | 9,4 |
| Fructose-bisphosphate aldolase A [ALDOA_HUMAN] | P04075 | LP1 | 5 | 79,67 | 117,70 | 13 | 39,4 | 32 | 8,09 | 8,5 |
| Glutamate dehydrogenase 1, mitochondrial [DHE3_HUMAN] | P00367 | CT6,7 | 1 | 60,75 | 292,31 | 22 | 61,4 | 43 | 7,80 | 7.3-7,5 |
| Glutathione S-transferase P [GSTP1_HUMAN] | P09211 | LP13 | 4 | 74,76 | 199,89 | 11 | 23,3 | 15,5 | 5,64 | 5,4 |
| Glyceraldehyde-3-phosphate dehydrogenase [G3P_HUMAN] | P04406 | KT3-5 | 1 | 59,10 | 205,37 | 15 | 36,0 | 33 | 8,46 | 9,5 |
| Heat shock protein beta-1 [HSPB1_HUMAN] | P04792 | LP12  CT18 | 7  1 | 75,61  43,90 | 127,81  54,81 | 9  3 | 22,8 | 20  20,5 | 6,40 | 5,4  5,4 |
| Heat shock 70 kDa protein 1A/1B [HSP71_HUMAN] | P08107 | KT16-18 | 1 | 57,41 | 669,21 | 30 | 70,0 | 64 | 5,66 | 5,7 |
| Heat shock protein HSP 90-alpha [HS90A_HUMAN] | P07900 | ET2 | 1 | 58,74 | 316,48 | 21 | 84,6 | 80 | 5,02 | 5,1 |
| Heat shock protein HSP 90-beta [HS90B_HUMAN] | P08238 | ET3 | 1 | 60,64 | 244,72 | 17 | 83,2 | 74 | 5,03 | 5.0 |
| Heterogeneous nuclear ribonucleoprotein K [HNRPK] | P61978 | SW8,9 | 4 | 54,64 | 234,46 | 23 | 50,9 | 64 | 5,54 | 5,1 |
| Heterogeneous nuclear ribonucleoprotein L [HNRPL] | P14866 | SW1 | 7 | 53,82 | 536,40 | 24 | 64,1 | 65 | 8,22 | 7,4 |
| Heterogeneous nuclear ribonucleoproteins C1/C2  [HSPC_HUMAN] | G3V4C1 | SW5 | 4 | 56,51 | 115,39 | 9 | 32,2 | 37 | 5,08 | 5.0 |
| Isoform 2 of Cytosol aminopeptidase [AMPL_HUMAN] | P28838-2 | ST4,5 | 1 | 55,94 | 76,61 | 12 | 52,7 | 49 | 6,74 | 7.0 |
| Isoform 2 of Enoyl-CoA delta isomerase 1, mitochondrial [ECI1_HUMAN] | P42126-2 | CT17 | 1 | 35,79 | 79,97 | 5 | 30,9 | 21 | 8,90 | 6,3 |
| Isoform 2 of Gelsolin [GELS_HUMAN] | P06396-2 | ET7 | 1 | 41,59 | 52,92 | 12 | 80,6 | 75 | 5,85 | 6,5 |
| Isoform 2 of Serine hydroxymethyltransferase, cytosolic SHMT1 [GLYC_HUMAN] | P34896-2 | LP7 | 5 | 61,26 | 117,24 | 10 | 49,0 | 44 | 7,69 | 7,8 |
| Isoform C of Prelamin-A/ [LMNA_HUMAN] | P02545-2 | SW2, 16  ET11 | 9  1 | 87,94 | 669,22 | 47 | 65,1 | 61 | 6,84 | 7,1 |
| Lamin-B2 [LMNB2_HUMAN] | Q03252 | LP4 | 5 | 85,50 | 1024,99 | 57 | 67,6 | 56 | 5,35 | 5,4 |
| Phosphoglycerate mutase 1 [PGAM1_HUMAN] | P18669 | CT15 | 1 | 57,87 | 117,60 | 10 | 28,8 | 21 | 7,18 | 7,3 |
| Proteasome subunit alpha type [G3V295_HUMAN] | G3V295 | CT16 | 1 | 64,53 | 116,79 | 9 | 22,8 | 20,5 | 8,32 | 6,7 |
| Proteasome subunit alpha type-2 [PSA2_HUMAN] | P25787 | KT9 | 1 | 51,71 | 98,07 | 6 | 25,9 | 23 | 7,43 | 7,5 |
| Protein disulfide isomerase family A, member 3, isoform CRA_b [G5EA52_HUMAN] | G5EA52 | ST9-12 | 1 | 69,69 | 324,92 | 26 | 54,9 | 48 | 6,86 | 5.6-5,8 |
| Protein phosphatase 1 regulatory subunit 7 [PP1R7_HUMAN] | Q15435 | SW6 | 4 | 46,39 | 70,89 | 10 | 41,5 | 44 | 4,91 | 4,6 |
| Rab GDP dissociation inhibitor beta OS=Homo sapiens [GDIB_HUMAN] | P50395 | ET18,19 | 1 | 43,82 | 52,23 | 10 | 50,6 | 41 | 6,47 | 6,8 |
| Ras-related protein Rab-14 [RAB14_HUMAN] | P61106 | KT10-12 | 1 | 70,70 | 38,79 | 7 | 23,9 | 22 | 6,21 | 5.5-7.5 |
| Retinal dehydrogenase 1 [AL1A1_HUMAN] | P00352 | LP10  ST3 | 4  1 | 81,24  44,91 | 684,77  42,55 | 35  12 | 54,8 | 46  49 | 6,73 | 6,8  7,1 |
| Rho GDP-dissociation inhibitor 1 OS=Homo sapiens GN=ARHGDIA PE=1 SV=3 - [GDIR1_HUMAN] | P52656 | CT19 | 1 | 41.67 | 26.89 | 4 | 23.2 | 19.5 | 5.11 | 4.5 |
| Septin 11, isoform CRA_b [D6RGI3_HUMAN] | D6RGI3 | CT5 | 1 | 12,47 | 19,48 | 1 | 49,0 | 41 | 6.96 | 6,3 |
| Serotransferrin [TRFE_HUMAN] | P02787 | ST18-22  ET8-10  CT1-4  PT1  KT19 | 1  1  1  1  1 | 47,28  59,74  68,34  72,78  51,72 | 134,78  175,77  589,83  1309,73  129,07 | 24  37  41  56  23 | 77,0 | 68  70-74  64  74  78 |  | 6.9-7.8  6,7  6,7  7,2  7,1 |
| Serum albumin [ALBU_HUMAN] | P02768 | ST14-ST17  ET4,5,6 | 1  1 | 64,37  69,95 | 151,68  241,34 | 27  27 | 69,3 | 48  80 | 6,28 | 5.9-6,1  6,2 |
| Vimentin [VIME_HUMAN] | P08670 | SW7,10, 14,19  LP6  PT2-5 | 13  4  1 | 85,19  73.18  68,24 | 414,65  112.49  376,38 | 34  22  26 | 53,6 | 50-52  62  35-37 | 5,12 | 5.00-5.1  4.8  4.9-5,1 |
| Non identified |  | SW4  ET1,12, 20,21,22  KT8 |  |  |  |  |  |  |  |  |

*, identification of two proteins in the same spot.
