# Supplementary material for: Autoantibody signatures defined by serological proteome analysis in sera from patients with cholangiocarcinoma
Source: J Transl Med. 2016 Jan 16;14:17. doi: 10.1186/s12967-015-0751-2 (PMC4715332; doi:10.1186/s12967-015-0751-2)
Supplement: Supplementary file 2 — 10.1186/s12967-015-0751-2 Identification of immunoreactive proteins in normal liver and in the five non-tumour counterparts adjacent to the cholangiocarcinoma. Spots with the NH abbreviation correspond to those indicated in Fig. 5 and stained by more than one-third of cholangiocarcinoma sera on normal liver. Spots with the CN. EN. KN. PN. or SN abbreviations, correspond to those indicated on the five gels in Fig. 7 and stained by the patient’s serum reacting with its own non-tumour liver proteins adjacent to the cholangiocarcinoma. [file 12967_2015_751_MOESM2_ESM.doc]

**Additional files 2: Table S2**. **Identification of immunoreactive proteins in normal liver and in the five non-tumour counterpart adjacent to the cholangiocarcinoma.** Spots with the NH abbreviation correspond to those indicated in Figure 7 and stained by more than one-third of cholangiocarcinoma sera on normal liver. Spots with the CN. EN. KN. PN. or SN abbreviations, correspond to those indicated on the five gels in Figure 6 and stained by the patient’s serum reacting with its own non-tumour liver proteins. Access numbers are from the Swiss-Prot database.

| **Protein Identification** | **Access number** | **Spot No** | **Number of reactive sera** | **Sequence coverage %** | **Score** | **Number of peptides matched** | **Molecular Weight (MW)** | | **Isoelectric Point (pI)** | |
| --- | --- | --- | --- | --- | --- | --- | --- | --- | --- | --- |
|  |  |  |  |  |  |  | **Theoretical** | **Observed** | **Theoretical** | **Observed** |
| 26S protease regulatory subunit 7 [PRS7_HUMAN] | P35998 | EN26 | 1 | 86.84 | 232.56 | 30 | 48.6 | 45 | 5.95 | 6.1 |
| 3-ketoacyl-CoA thiolase. mitochondrial [THYM_HUMAN] | P42765 | NH8, 9  PN13  SN9* | 6  1  1 | 88.66  69.77  56.68 | 1303.69  172.34  115.46 | 34  17  10 | 41.9  41.9  41.9 | 43  43  30 | 8.09  8.09  8.09 | 9.5  8.8  7.6 |
| 3-mercaptopyruvate sulfurtransferase [THTM_HUMAN] | P25325 | PN26 | 1 | 79.12 | 369.13 | 15 | 33.2 | 32 | 6.60 | 7.6 |
| 4-hydroxyphenylpyruvate dioxygenase [HPPD_HUMAN] | P32754 | EN40 | 1 | 84.99 | 803.01 | 29 | 44.9 | 40 | 7.01 | 6.9 |
| 60 kDa heat shock protein. mitochondrial [CH60_HUMAN] | P10809 | SN7*  EN14,15  CN9 | 1  1  1 | 53.05  90.75  75.57 | 323.22  1153.25  504.72 | 22  56  30 | 61.0  61.0  61.0 | 42  58  56 | 5.87  5.87  5.87 | 5.1  5.4  6.0 |
| 78 kDa glucose-regulated protein [GRP78_HUMAN] | P11021 | EN17 | 1 | 73.09 | 997.71 | 55 | 72.3 | 72 | 5.16 | 5.0 |
| Acetyl-CoA acetyltransferase cytosolic [THIC_HUMAN] | Q9BWD1 | PN18 | 1 | 70.53 | 131.17 | 14 | 41.3 | 38 | 6.92 | 7.6 |
| Acetyl-CoA acetyltransferase. mitochondrial[THIL_HUMAN] | P24752 | PN12  KN14  NH18 | 1  1  4 | 84.07  50.08  69.79 | 404.46  83.83  852.32 | 29  10  22 | 45.2  45.2  45.2 | 40  37  40 | 8.85  8.85  8.85 | 9.6  8.8  8.9 |
| Aconitate hydratase. Mitochondrial [ACON_HUMAN] | Q99798 | NH16 | 5 | 28.21 | 51.74 | 9 | 85.4 | 75 | 7.61 | 7.5 |
| Actin. cytoplasmic 1 [ACTB_HUMAN] | P60709 | PN19 | 1 | 71.20 | 224.44 | 7 | 41.7 | 45 | 5.48 | 7.0 |
| Actin-related protein 2/3 complex subunit 2 [ARPC2_HUMAN] | O15144 | PN25 | 1 | 88.33 | 337.89 | 23 | 34.3 | 28 | 7.36 | 7.8 |
| Aflatoxin B1 aldehyde reductase member 3  [ARK73_HUMAN] | O95154 | EN35 | 1 | 58.01 | 1164.97 | 13 | 37.2 | 34 | 7.15 | 6.9 |
| Alcohol dehydrogenase [NADP(+)][AK1A1_HUMAN] | P14550 | PN17 | 1 | 75.08 | 694.55 | 27 | 36.5 | 37 | 6.79 | 7.7 |
| Alcohol dehydrogenase 4 [[ADH4_HUMAN]] | P08319 | EN28 | 1 | 85.79 | 592.75 | 27 | 40.2 | 37 | 7.94 | 9.8 |
| Aldehyde dehydrogenase. mitochondrial [F8w0A9_HUMAN] | F8W0A9 | NH14 | 4 | 62.07 | 169.46 | 18 | 40.8 | 39 | 6.25 | 5.6 |
| Aldo-keto reductase family 1 member C2 [AK1C2_HUMAN] | P52895 | EN33,34 | 1 | 88.54 | 779.03 | 16 | 36.7 | 34 | 7.49 | 7.4-7.9 |
| Aldo-keto reductase family 1 member C4 [AK1C4_HUMAN] | P17516 | EN36 | 1 | 79.88 | 638.92 | 18 | 37.0 | 31 | 6.93 | 8.8-9.5 |
| Alpha-enolase [ENOA_HUMAN] | P06733 | PN9,11  EN19* | 1  1 | 87.33  84.40 | 560.61  318.46 | 38  30 | 47.1 | 47  42 | 7.39 | 9.5  6.5 |
|  |  |  |  |  |  |  |  |  |  |  |
| Aminoacylase-1 [ACY1_HUMAN] | Q03154 | PN20 | 1 | 88.97 | 1125.39 | 28 | 45.9 | 43 | 6.18 | 6.8 |
| Annexin A2 [ANXA2_HUMAN] | P07355 | EN32 | 1 | 83.78 | 435.60 | 34 | 38.6 | 30 | 7.75 | 8.4 |
| Arginase-1 [ARGI1_HUMAN] | P05089 | NH1, 2  PN15  SN10 | 7  1  1 | 76.09  75.16  45.52 | 716.78  450.85  46.07 | 29  25  6 | 34.7  34.7  34.7 | 36  38  33 | 7.21  7.21  7.21 | 7.2  8.1-8.3  7.3 |
| ATP synthase subunit beta. mitochondrial [ATPB_HUMAN] | P06576 | PN22-24  SN6,7* | 1  1 | 87.15  78.64 | 2442.19  1158.28 | 50  29 | 56.5  56.5 | 47-51  42-47 | 5.40  5.40 | 5.5-5.8  5.1-5.2 |
| Beta-enolase [ENOB_HUMAN] | P13929 | EN27  KN16 | 1  1 | 78.57  61.52 | 372.63  130.46 | 25  13 | 47.0  47.0 | 40  45 | 7.71  7.71 | 8.5  8. 8 |
| Bifunctional ATP-dependent dihydroxyacetone kinase/FAD-AMP lyase (cyclizng) [DHAK_HUMAN] | Q3LXA3 | NH13  EN8 | 5  1 | 50.78  68.52 | 111.93  437.87 | 14  33 | 58.9  58.9 | 56  58 | 7.49  7.49 | 7.8  7.5 |
| Carbamoyl-phosphate synthase [ammonia]. mitochondrial [CPSM_HUMAN] | P31327 | KN18 | 1 | 19.13 | 77.54 | 10 | 164.8 | 88 | 6.74 | 6.2 |
| Carbonic anhydrase 1 [CAH1_HUMAN] | P00915 | NH5 | 4 | 67.82 | 540.26 | 18 | 28.9 | 29 | 7.12 | 6.8 |
| Catalase [CATA_HUMAN] | P04040 | PN1- 4  EN10 | 1  1 | 75.71  64.52 | 954.70  515.60 | 34  34 | 59.7  59.7 | 64  57 | 7.39  7.39 | 8.5-9.8  7.5 |
| D-beta-hydroxybutyrate dehydrogenase [E9PCG9_HUMAN] | E9PCG9 | SN2 | 1 | 57.03 | 58.36 | 1 | 29.0 | 66 | 7.78 | 8.0 |
| Delta(3.5)-Delta(2.4)-dienoyl-CoA isomerase. mitochondrial [ECH1_HUMAN] | Q13011 | PN27, 28  NH4 | 1  4 | 86.89  90.24 | 654.30  631.36 | 26  28 | 35.8  35.8 | 30-31  29 | 8.00  8.00 | 7.0-7.2  6.6 |
| Delta-1-pyrroline-5-carboxylate dehydrogenase. mitochondrial [AL4A1_HUMAN] | P30038 | NH12 | 4 | 55.95 | 214.52 | 11 | 61.7 | 54 | 8.07 | 7.6 |
| Dihydrolipoyl dehydrogenase. mitochondrial [DLDH_HUMAN] | P09622 | CN4 | 1 | 66.01 | 408.13 | 19 | 54.1 | 56 | 7.85 | 7.9 |
| Electron transfer flavoprotein subunit alpha. mitochondrial [ETFA_HUMAN] | P13804 | NH15 | 5 | 84.38 | 782.75 | 24 | 35.1 | 29 | 8.38 | 7.6 |
| Elongation factor 2 [EF2_HUMAN] | P13639 | EN1. 2 | 1 | 59.91 | 559.52 | 37 | 95.3 | 90 | 6.83 | 7.4-7.5 |
| Epoxide hydrolase 1 [HYEP_HUMAN] | P07099 | SN5  CN1 | 1  1 | 51.43  29.45 | 60.83  47.11 | 3  4 | 52.9  52.9 | 45  50 | 7.25  7.25 | 8.7  9.5 |
| Estradiol 17-beta-dehydrogenase 8 [DHB8_HUMAN] | Q92506 | NH3 | 5 | 53.26 | 109.01 | 12 | 27.0 | 29 | 6.54 | 6.9 |
| Fructose-1.6-bisphosphatase 1 [F16P1_HUMAN] | P09467 | NH10*  PN16 | 5  1 | 40.53  66.86 | 51.28  855.44 | 8  24 | 36.8  36.8 | 26  38 | 6.99  6.99 | 7.2  8.1 |
| Fructose-bisphosphate aldolase B [ALDOB_HUMAN] | P05062 | NH10*  CN11, 13  EN29-31  KN15  PN14 | 5  1  1  1  1 | 59.34  62.64  79.12  76.10  76.65 | 217.47  403.60  1271.63  401.75  373.89 | 16  11  21  17  17 | 39.4  39.4  39.4  39.4  39.4 | 26  33  35  37  37 | 7.87  7.87  7.87  7.87  7.87 | 7.2  6.9-7.8  8.1-9.9  9.3  8.8 |
| Fumarylacetoacetate hydrolase domain-containing protein 2A [FAH2A_HUMAN] | Q96GK7 | SN9* | 1 | 60.19 | 326.48 | 14 | 34.6 | 30 | 8.24 | 7.6 |
| Glyceraldehyde-3-phosphate dehydrogenase [G3P_HUMAN] | E7EUT4 | NH11  NH6  KN13 | 1  7  1 | 73.72  72.35  71.33 | 164.52  210.13  131.57 | 16  13  12 | 31.5  31.5  31.5 | 32  29  28 | 7.61  7.61  7.61 | 7.6  7.3  7.5 |
| Glycine amidinotransferase. mitochondrial [GATM_HUMAN] | P50440 | EN21-25 | 1 | 89.26 | 667.43 | 31 | 44.9 | 40 | 7.06 | 6.9-7.2 |
| Haloacid dehalogenase-like hydrolase domain-containing protein 3 [HDHD3_HUMAN] | Q9BSH5 | PN29 | 1 | 69.32 | 246.75 | 9 | 28.0 | 26 | 6.71 | 8.5 |
| Heat shock cognate 71 kDa protein [HSP7C_HUMAN] | P11142 | EN16 | 1 | 72.91 | 729.39 | 42 | 70.9 | 68 | 5.52 | 5.5 |
| Heterogeneous nuclear ribonucleoprotein L [HNRPL_HUMAN] | P14866 | EN7 | 1 | 44.82 | 138.15 | 13 | 64.1 | 60 | 8.22 | 7.5 |
| Hydroxymethylglutaryl-CoA synthase. mitochondrial [HMCS2_HUMAN] | P54868 | PN10 | 1 | 28.94 | 53.80 | 6 | 56.6 | 45 | 8.16 | 9.4 |
| Isoform 2 of Glycine amidinotransferase. mitochondrial [GATM_HUMAN] | P50440-2 | EN19*, 20 | 1 | 84.40 | 358.99 | 28 | 44.9 | 42 | 7.06 | 6.5-6.6 |
| Isoform A2 of Heterogeneous nuclear ribonucleoproteins A2/B1 [ROA2_HUMAN] | P22626-2 | CN12 | 1 | 42.23 | 35.71 | 2 | 36.0 | 32 | 8.65 | 7.6 |
| Liver carboxylesterase 1 [E9PAU8_HUMA] | E9PAU8 | KN11, 12  EN9 | 1  1 | 58.30  57.95 | 226.63  215.64 | 20  23 | 62.4  62.4 | 50  65 | 6.60  6.60 | 6.8  6.6 |
| Liver carboxylesterase 1 [EST1_HUMAN] | P23141 | EN11, 12 | 1 | 67.02 | 827.23 | 40 | 62.5 | 57 | 6.60 | 6.5 |
| Methylmalonate-semialdehyde dehydrogenase [acylating]. mitochondrial [MMSA_HUMAN] | Q02252 | PN6 | 1 | 65.05 | 318.18 | 32 | 57.8 | 56 | 8.50 | 9.8 |
| Methylmalonyl-CoA mutase. mitochondrial [MUTA_HUMAN] | P22033 | EN3 | 1 | 39.20 | 167.64 | 18 | 83.1 | 79 | 6.93 | 6.4 |
| Neutral alpha-glucosidase AB [GANAB_HUMAN] | Q14697 | KN19 | 1 | 33.69 | 142.41 | 10 | 106.8 | 88 | 6.14 | 5.9 |
| Non-specific lipid-transfer protein [NLTP_HUMAN] | P22307 | EN18 | 1 | 57.04 | 808.50 | 31 | 59.0 | 45 | 6.89 | 6. 4 |
| Phosphoenolpyruvate carboxykinase [GTP], mitochondrial [PCKGM_HUMAN] | Q16822 | EN5 | 1 | 60,47 | 835,15 | 29 | 70,7 | 65 | 7,62 | 7.8 |
| Phosphoglycerate mutase 1 [PGAM1_HUMAN] | P18669 | PN30 | 1 | 71,26 | 591,48 | 14 | 28,8 | 26 | 7,18 | 7.5 |
| Phosphoserine aminotransferase [SERC_HUMAN] | Q9Y617 | KN14* | 1 | 72,43 | 197,14 | 19 | 40,4 | 37 | 7,66 | 8.8 |
| Isoform C of Prelamin-A/ [LMNA_HUMAN] | P02545-2 | NH17  EN6  CN5-7 | 4  1  1 | 45,45  57,69  40,03 | 81,94  130,72  106,54 | 14  24  11 | 65,1  65,1  65,1 | 43  60  56 | 6,84  6,84  6,84 | 6.1  7.2  6.8-7.2 |
| Prelamin-A/C [LMNA_HUMAN] | P02545 | KN10 | 1 | 29,07 | 36,42 | 8 | 74,1 | 72 | 7,02 | 7.0 |
| Protein KRT17P1 - [A8MW45_HUMAN] | A8MW45 | CN3 | 1 | 8,84 | 22,57 | 1 | 48,5 | 56 | 4,89 | 8.2 |
| Retinal dehydrogenase 1 [AL1A1_HUMAN] | P00352 | PN8  CN2 | 1  1 | 84,23  8,84 | 642,03 | 38  1 | 54,8  48,5 | 57  56 | 6,73  4,89 | 8.2  8.5 |
| Serotransferrin [TRFE_HUMAN] | P02787 | KN3-9,17 | 1 | 71,06 | 729,24 | 51 | 77,0 | 73 | 7,12 | 6.9-8.0 |
| Serum albumin [ALBU_HUMAN] | P02768 | CN10  EN4  SN4 | 1  1  1 | 35,14  53,53  71,92 | 67,46  143,87  347,52 | 9  18  26 | 69,3  69,3  69,3 | 48  79  47 | 6,28  6,28  6,28 | 6.1  6,4  6.9 |
| Short/branched chain specific acyl-CoA dehydrogenase, mitochondrial [ACDSB_HUMAN] | P45954 | PN21 | 1 | 54,86 | 222,45 | 22 | 47,5 | 41 | 6,99 | 6.8 |
| Short-chain specific acyl-CoA dehydrogenase, mitochondrial [ACADS_HUMAN] | P16219 | SN8 | 1 | 58,25 | 179,30 | 13 | 44,3 | 35 | 7,99 | 7.1 |
| Stress-70 protein [GRP75_HUMAN] | P38646 | SN3 | 1 | 18,26 | 22,78 | 4 | 73,6 | 66 | 6,16 | 7.8 |
| S-methyl-5'-thioadenosine phosphorylase [MTAP_HUMAN] | Q13126 | NH7 | 5 | 84,81 | 117,57 | 15 | 31,2 | 26 | 7,18 | 7,6-7.7 |
| Thiosulfate sulfurtransferase [THTR_HUMAN] | Q16762 | EN37,38 | 1 | 82,15 | 1459,82 | 18 | 33,4 | 32 | 7,25 | 7,4 |
| UTP--glucose-1-phosphate uridylyltransferase [UGPA_HUMAN] | Q16851 | PN5,7 | 1 | 79,92 | 1018,23 | 48 | 56,9 | 57 | 8,15 | 8,8-9.5 |
| Non identified |  | CN8  EN13,39  KN1,2,20  SN1 |  |  |  |  |  |  |  |  |

* Two identifications in the same spots with high probability.
