# Supplementary material for: Autoantibody signatures defined by serological proteome analysis in sera from patients with cholangiocarcinoma
Source: J Transl Med. 2016 Jan 16;14:17. doi: 10.1186/s12967-015-0751-2 (PMC4715332; doi:10.1186/s12967-015-0751-2)
Supplement: Supplementary file 3 — 10.1186/s12967-015-0751-2 Gene Ontology distribution of proteins recognized by CC sera, according to the protein class. [file 12967_2015_751_MOESM3_ESM.docx]

**Additional file 3: Table S3. Gene Ontology distribution of proteins recognized by CC sera, according to the protein class**.

|  | CCSW1 | CCLP1 | Tumour part | Adjacent non- tumour part | Normal liver |
| --- | --- | --- | --- | --- | --- |
| Cytoskeletal protein | 27.3% | 30.0% | 10.0% | 6.3% | 4.8% |
| Structural protein | 18.2% | 30.0% | - | 6.3% | 4.8% |
| Nucleic acid binding | 27.3% | - | 10.0% | 6.3% | - |
| Transfer/carrier | - | - | 20.0% | 6.3% | - |
| Transporter | - | - | 10.0% | 6.3% | - |
| Chaperone | 9.1% | 20.0% | - | 6.3% | - |
| Enzyme modulator | 9.1% | - | - | - | - |
| Oxydoreductase | 9.1% | 10.0% | - | 12.5% | 28.6% |
| Transferase | - | 10.0% | - | 12.5% | 23.8% |
| Hydrolase | - | - | 20.0% | 18.8% | 9.5% |
| Isomerase | - | - | - | - | 4.8% |
| Ligase | - | - | - | - | 4.8% |
| Lyase | - | - | 10.0% | 12.5% | 14.3% |
| Phosphatase | - | - | - | - | 4.8% |
| Protease | - | - | 10.0% | - | - |
| Receptor | - | - | 10.0% | 6.3% | - |
