# Supplementary material for: Autoantibody signatures defined by serological proteome analysis in sera from patients with cholangiocarcinoma
Source: J Transl Med. 2016 Jan 16;14:17. doi: 10.1186/s12967-015-0751-2 (PMC4715332; doi:10.1186/s12967-015-0751-2)
Supplement: Supplementary file 4 — 10.1186/s12967-015-0751-2 Gene Ontology distribution of proteins recognized by CC sera, according to the pathway involved. [file 12967_2015_751_MOESM4_ESM.docx]

**Additional file 4: Table S4. Gene Ontology distribution of proteins recognized by CC sera, according to the pathway involved**.

|  | CCSW1 | CCLP1 | Tumour part | Adjacent non- tumour part | Normal liver |
| --- | --- | --- | --- | --- | --- |
| Fructose galactose metabolism | - | 6.3% | - | 20.0% | 12.5% |
| Glycolysis | - | 6.3% | 9.1% | 40.0% | 25.0% |
| ATP synthesis | - | - | 9.1% | 20.0% | - |
| Fas signalling pathway | - | 6.3% | - | 20.0% | 12.5% |
| 5-hydroxytryptamine degradation | 12.5%% | - | - | - | 12.5% |
| Alzheimer's disease-presenilin pathway | 12.5% | 6.3% | 9.1% | - | - |
| Angiogenesis | - | 6.3% | - | - | - |
| Apoptosis signalling pathway | - | 6.3% | - | - | - |
| Cadherin signalling pathway | 12.5% | 6.3% | 9.1% | - | - |
| Cytoskeletal regulation by Rho GTPase | 12.5% | 6.3% | 9.1% | - | - |
| Gonadotrophin releasing hormone receptor pathway | - | - | 9.1% | - | - |
| Huntington's disease | 12.5% | 6.3% | 9.1% | - | 12.5% |
| Inflammation mediated by chemokine and cytokine signalling pathway | 12.5% | 6.3% | 9.1%% | - | - |
| Integrin signalling pathway | 12.5% | 6.3% | 9.1% | - | - |
| Nicotinic acetylcholine receptor signalling pathway | 12.5% | 6.3% | 9.1% | - | - |
| Parkinson's disease | - | 6.3% | 9.1% | - | - |
| Purine metabolism | - | - | - | - | 12.5% |
| Serine glycine biosynthesis | - | 6.3% | - | - | - |
| TCA cycle | - | - | - | - | 12.5% |
| VEGF-signalling pathway | - | 6.3% | - | - | - |
| P38 MAPK pathway | - | 6.3% | - | - | - |
