# Supplementary figures and images for: Autoantibody signatures defined by serological proteome analysis in sera from patients with cholangiocarcinoma
Source: J Transl Med. 2016 Jan 16;14:17. doi: 10.1186/s12967-015-0751-2 (PMC4715332; doi:10.1186/s12967-015-0751-2)

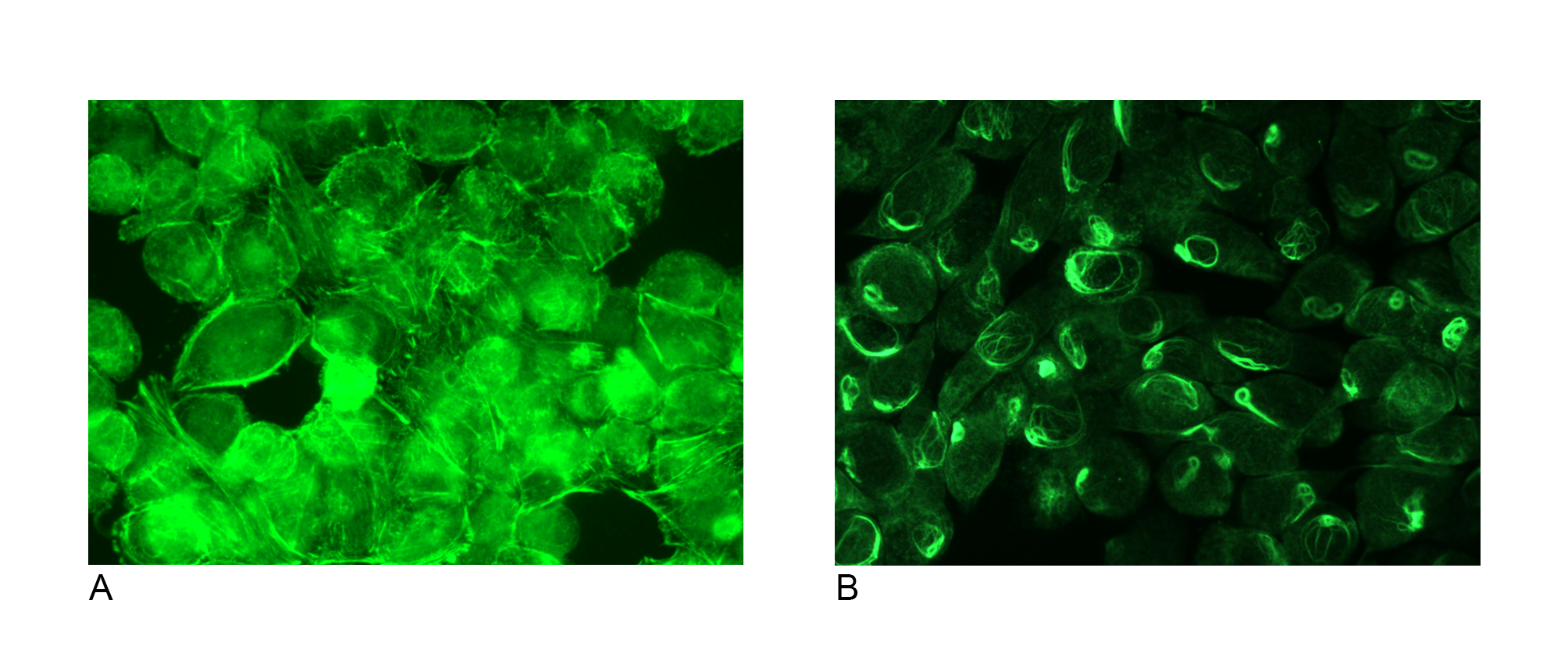

Supplement: Supplementary file 5 — 10.1186/s12967-015-0751-2 Anti-actin and anti-vimentin evaluation using immunofluorescence on colchicine-treated Hep2 cells. Sera positive by MS for anti-actin or anti-vimentin autoantibodies were tested by indirect immunofluorescence; (A) typical pattern of actin-cable strongly stained by anti-actin antibody; (B) typical pattern given by anti-vimentin antibody, vimentin colchicine-treated collapses into perinuclear coils. [file 12967_2015_751_MOESM5_ESM.tiff]
